# Protocol for *in vivo* immune cell analysis in subcutaneous murine tumor models using advanced flow cytometry

**DOI:** 10.1016/j.xpro.2024.103505

**Published:** 2025-01-15

**Authors:** Katrine Ingelshed, Marit M. Melssen, Diana Spiegelberg

**Affiliations:** 1 Department of Immunology, Genetics and Pathology, Uppsala University, 75185 Uppsala, Sweden; 2 Department of Surgical Sciences, Uppsala University, 75185 Uppsala, Sweden

**Keywords:** cell separation/fractionation, flow cytometry, cancer, immunology, model organisms

## Abstract

Here, we present a protocol for guiding tissue preparation and flow cytometric analysis in subcutaneous murine tumor models and secondary lymphoid organs. We describe steps for dissociating tumors, spleens, and lymph nodes to obtain single-cell suspensions. We then detail procedures for immune cell staining and analysis and gating strategies including the use of fluorescence-minus-one controls (FMOs). This approach provides valuable insights into the impact of cancer therapies on the tumor and systemic immune response.

For complete details on the use and execution of this protocol, please refer to Ingelshed et al.^1^

## Highlights

• Steps on dissociating tumors, spleens, and lymph nodes to obtain single-cell suspensions
• Instructions for preparing antibody cocktails for immune cell staining and FMO controls
• Guidance on gating strategies to assess systemic and local tumor-related immune responses
• Steps for statistical analysis using multiple regression to adjust for tumor size

## Abstract (editor highlights)

Publisher’s note: Undertaking any experimental protocol requires adherence to local institutional guidelines for laboratory safety and ethics.

## Graphical abstract

**Figure:**
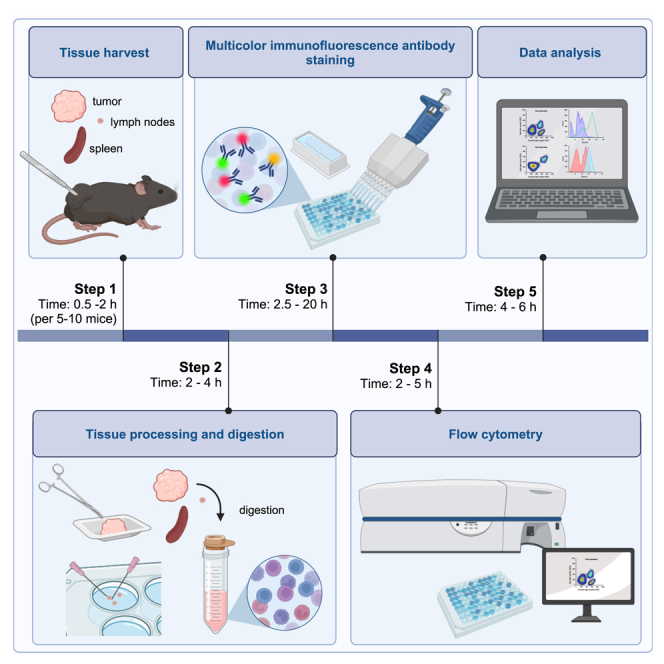

## Before you begin

In recent years there has been a tremendous improvement in the treatment of solid tumors, due to the discovery and approval of immune therapies.^2^^,^^3^ These successes have highlighted the importance of the tumor microenvironment, and the magnitude of the systemic immune response in the fight against cancer.^4^^,^^5^ To evaluate the immune cell composition of the local microenvironment and systemic immune response in murine tumor models through flow cytometry, it is vital to obtain proper single cell suspensions of the tumor and secondary lymphoid organs.^6^ Many immune cell populations are however, difficult to extract from tissues as these cells tend to firmly attach themselves to other cells and structures.^7^ In this protocol we describe how to process tumor tissues, spleens and lymph nodes to prepare single cell suspensions and extract lymphoid and myeloid immune cell populations such as naïve, activated, effector and memory subsets of CD8+ and CD4+ T cells, regulatory T cells (Tregs), CD11b- NK cells, mature B cells, dendritic cell (DC) subsets, M1- and M2-like macrophages, polymorphonuclear leukocytes (PMNs) and myeloid derived suppressor cells (MDSCs). Further, we thoroughly describe the process of staining and analyzing the samples, compensation controls and FMO controls. The protocol described has been developed specifically for murine subcutaneous tumor models. We recommend to use mice older than 8 weeks and younger than 12 weeks at the start of the experiment, further to achieve robust statistical significance, use a minimum of 5 animals per experimental group.

### Institutional permissions

In this study, all animal experiments were conducted in accordance with Swedish legislation and with the required permissions from the Uppsala Committee of Animal Research Ethics. Any experiments on mice must be performed in accordance with relevant institutional and national guidelines and regulations. Ensure that permission exists from the relevant institutions prior to starting the experiments.

### Prepare tissue harvest buffers

**Timing: 1 h**

1. Ensure availability of all reagents listed in the key resources table and materials and equipment section.

***Note:*** We recommend to prepare reagents in advance for better time management on the day of the experiment.

2. Prepare the tissue harvest buffers:
  a. Prepare the Stock digest solution according to the materials and equipment section and as described in the step-by-step method details section, step 5.
    i. Store the prepared buffer at 2°C–8°C or on ice.
      ***Note:*** The Complete Digest Solution has to be prepared fresh before each experiment. It can be prepared up to 24 h prior to the experiment, provided it is kept cold at all steps.
  b. Prepare the Awesome Wash Buffer according to the materials and equipment section, also described in the step-by-step method details, step 6.
    i. Store the prepared buffer at 2°C–8°C or on ice.
      ***Note:*** The preparation of the Stock Digest Solution and the Awesome Wash Buffer can be completed the day before starting the experiment.
  c. Prepare the Red Blood Cell Lysis buffer by diluting one part of the 10× solution with nine parts deionized water. See also the materials and equipment section and calculation example in the step-by-step method details section, step 22.
    i. Store the prepared buffer at 2°C–8°C or on ice.
      ***Note:*** The Red Blood Cell (RBC) Lysis Buffer can be diluted to 1× the day before the experiment start and stored at 2°C–8°C.

### Prepare extracellular antibody cocktails and controls

**Timing: 2–3 h**

3. Prepare the Staining Buffer from the Brilliant Stain Buffer according to the materials and equipment section and the detailed description in the step-by-step method details section, step 11, with calculation examples in step 10.

***Note:*** The Staining Buffer can be prepared the day before the experiment start and stored at 2°C–8°C.

4. Prior to starting the experiment, prepare the extracellular antibody cocktails, as described in detail in the step-by-step method details section, steps 7–17.

***Note:*** The cocktails can be prepared the day before the tissue harvest and stored at 2°C–8°C.

### Prepare fix/perm and perm buffer

**Timing: 10 min**

5. Prepare the Fix/Perm buffer according to the materials and equipment section and the step-by-step method details section, step 80 f, with a detailed calculation example.
  a. The buffer lasts for 24 h if stored at 2°C–8°C.
6. Prepare the Perm buffer according to the materials and equipment section and the step-by-step method details section, step 80 f, with a detailed calculation example.
  a. The buffer lasts for 24 h if stored at 2°C–8°C.

***Note:*** These buffers last for 24 h and will be used on the day of the *in vivo* endpoint and the day after. The Fix/Perm and Perm Buffers are therefore preferably prepared the same day that the antibody staining is started.

### Prepare intracellular antibodies

**Timing: 10 min**

7. Prepare the antibodies in Perm Buffer as described in detail in the step-by-step method details section, steps 83–85, just before performing intracellular staining.

### Prepare bead-based compensation controls

**Timing: 30 min**

8. Prepare the bead-based compensation controls as described in detail in the step-by-step method details section, steps 92–103.

***Note:*** Stain the beads with the antibodies used (all colors except BV510/the L/D dye), just before starting the compensation in the flow cytometer.

## Key resources table

**Table:** 

REAGENT or RESOURCE	SOURCE	IDENTIFIER
**Antibodies**		

Anti-CD11b, BV510 conjugated (clone M1/70), (1:400)	BD Biosciences	Cat# 562950; RRID:AB_2737913
Anti-CD45, BV786 conjugated (clone 30-F11), (1:300)	BD Biosciences	Cat# 564225; RRID:AB_2716861
Anti-CD3, PerCPCy5.5 conjugated (clone 145-2C11), (1:50)	Thermo Fisher Scientific	Cat# 45-0031-82; RRID:AB_1107000
Anti-B220, PE conjugated (clone RA3-6B2), (1:100)	BioLegend	Cat# 103208; RRID:AB_312992
Anti-CD8a, APC conjugated (clone 53–6.7), (1:200)	BioLegend	Cat# 100712; RRID:AB_312750
Anti-CD4, BV605 conjugated (clone RM4-5), (1:200)	BioLegend	Cat# 100548; RRID:AB_2563054
Anti-CD25, PECy7 conjugated (clone PC61), (1:200)	BD Biosciences	Cat# 561780; RRID:AB_10893596
Anti-CD44, BV421 conjugated (clone IM7), (1:200)	BioLegend	Cat#103040; RRID:AB_10895752
Anti-CD62L, BV711 conjugated (clone Mel-14), (1:200)	BioLegend	Cat# 104445; RRID:AB_2564215
Anti-CD335 (NKp46), FITC conjugated (clone 29A1.4), (1:200)	BioLegend	Cat#137606; RRID:AB_2149150
Anti-Foxp3, Biotin conjugated (clone FJK-16s), (1:100)	Thermo Fisher Scientific	Cat# 13-5773-82; RRID:AB_763540
Anti-Arginase I, Alexa 488 conjugated (clone A1exF5), (1:100)	Thermo Fisher Scientific	Cat# 53-3697-82, RRID:AB_2734831
Anti-CD86, PE conjugated (clone GL-1), (1:200)	BioLegend	Cat# 105007, RRID:AB_313150
Anti-F4/80, PerCP-Cy5.5 conjugated (clone BM8), (1:100)	BioLegend	Cat# 123128, RRID:AB_893484
Anti-I-A/I-E, BV421 conjugated (clone M5/114.15.2), (1:200)	BioLegend	Cat# 107632, RRID:AB_2650896
Anti-CD11c, PE-Cy7 conjugated (clone HL3), (1:200)	BD Biosciences	Cat# 558079, RRID:AB_647251
Anti-Ly6G, BV605 conjugated (clone 1A8), (1:200)	BioLegend	Cat# 127639, RRID:AB_2565880
Anti-Ly6C, APC/Fire750 conjugated (clone HK1.4), (1:200)	BioLegend	Cat# 128046, RRID:AB_2616731
Anti-CD11b, BV711 conjugated (clone M1/70), (1:400)	BD Biosciences	Cat# 563168, RRID:AB_2716860
Anti-CD19, BV510 conjugated (clone 6D5), (1:200)	BioLegend	Cat# 115546, RRID:AB_2562137
Anti-TCR β chain, BV510 conjugated (clone H57-597), (1:200)	BD Biosciences	Cat# 563221, RRID:AB_2738078
Anti-CD335 (NKp46), BV510 conjugated (clone 29A1.4), (1:200)	BioLegend	Cat# 137623, RRID:AB_2563290
Anti-CD16/CD32, Fc block (clone 2.4G2), (1:400)	BD Biosciences	Cat# 553141, RRID:AB_394656
Anti-CD8a, Alexa 488 conjugated (clone 53-6.7), (1:200)	BioLegend	Cat# 100723, RRID:AB_389304
Anti-Siglec H, PE conjugated (clone 551), (1:100)	BioLegend	Cat# 129606, RRID:AB_2189147
Anti-PD1, BV421 conjugated (clone 29F.1A12), (1:200)	BioLegend	Cat# 135218, RRID:AB_2561447

**Chemicals, peptides, and recombinant proteins**		

Streptavidin, APC/Fire750 conjugated, (1:100)	BioLegend	Cat# 405250
10× red blood cell lysis buffer	BioLegend	Cat # 420302
Liberase	Roche	Cat # 05401127001
DNase I (bovine pancreas)	Roche	Cat # 11284932001
DMEM	Sigma-Aldrich	Cat #D5671
Heat-inactivated fetal bovine serum (FBS)	HyClone	Cat # SV30160.03
Dextrose (D-glucose)	Sigma-Aldrich	Cat #G8270
MEM non-essential amino acids	Gibco	Cat # 11140-050
MEM essential amino acids	Gibco	Cat # 11130-036
Sodium pyruvate	Gibco	Cat # 11360-070
L-glutamine	Gibco	Cat # 25030081
HEPES	Gibco	Cat # 15630-056
Bovine serum albumin (BSA)	Sigma-Aldrich	Cat # A9647
PBS tablets pH 7.4	Medicago	Cat # 09-9400-100
Invitrogen Brilliant stain buffer	Thermo Fisher Scientific	Cat # 00-4409-42
β-mercaptoethanol	Sigma-Aldrich	Cat #M3148
Gentamicin	Gibco	Cat # 15750060
EDTA	Amresco	Cat #E177

**Critical commercial assays**		

CD45 MicroBeads, mouse	Miltenyi Biotec	Cat# 130-052-301, RRID:AB_2877061
CD31 MicroBeads, mouse	Miltenyi Biotec	Cat# 130-097-418, RRID:AB_2814657
Live/Dead Fixable Aqua Dead Cell Stain Kit	Thermo Fisher Scientific	Cat#: L34966
eBioscience Foxp3/transcription factor staining buffer set	Thermo Fisher Scientific	Cat # 00-5523-00
UltraComp eBeads Plus compensation beads	Thermo Fisher Scientific	Cat # 01-3333-42

**Software and algorithms**		

FlowJo v.10	FlowJo	RRID:SCR_008520
R version 4.2.2 (optional: RStudio version 2024.04)	The R Foundation	RRID:SCR_001905

**Experimental models and cell lines**		

BALB/cAnNRj, 8–12 weeks, male and female	Janvier Labs	N/A
CT26.WT	ATCC	Cat # CRL-2638

**Equipment**		

BD LSR II	BD Biosciences	N/A
Cytoflex LX	Beckman Coulter	N/A
AutoMACS cell separator	Miltenyi Biotec	N/A

***Note:*** In the example analysis utilized in this protocol, the CT26.WT tumor model was implanted in BALB/cAnNRj. However, other subcutaneous tumor models, for example B16-F10 in C57BL/6, can be processed and assessed in the same way.

## Materials and equipment

**Table:** Stock digest solution

Reagent	Final concentration	Amount
DMEM	N/A	500 mL
Fetal bovine serum	2%	10 mL
HEPES (1 M)	15 mM	7.5 mL
L-glutamine (200 mM)	2 mM	5 mL
Sodium pyruvate (100 mM)	1 mM	5 mL
Non-essential Amino-Acids (100×)	1×	5 mL
Essential Amino-Acids (50×)	1×	10 mL
β-mercaptoethanol (50 mM)	0.05 mM	500 μL
**Total**	**N/A**	**543 mL**

Store sterile buffer at 2°C–8°C for up to one month.

**CRITICAL:** Check and adjust the pH of the Stock Digest Solution to 7.4 before use.

***Note:*** The Stock Digest Solution is sterile due to the use of sterile components. Filtering may prevent contamination during extended storage or incubations, though it's not needed for prompt or semi-sterile digestion.

***Note:*** The Stock Digest Solution does not require additional L-glutamine and Sodium pyruvate if the DMEM is already supplemented by the vendor.

***Note:*** It is not required to remove the total amount of volume for all added reagents combined (43 mL) from the stock 500 mL of DMEM when preparing the Stock Digest Solution.

***Note:*** It is possible to add 10 μg/mL Gentamicin to the Stock Digest Solution to reduce the chance for contamination.

***Note:*** β-mercaptoethanol prevents the build-up of oxygen radicals and therefore improves murine T cell viability. However, for an end-point flow cytometry approach this is not crucial and can be excluded from the buffer if desired.

**Table:** Complete digest solution

Reagent	Final concentration	Amount
Stock Digest Solution	N/A	100 mL
DNase I (10 mg/mL)	100 μg/mL	1 mL
Liberase (10 mg/mL)	75 μg/mL	0.750 mL
**Total**	**N/A**	**101.75 mL**

Store sterile buffer at 2°C–8°C or on ice for up to 24 h.

***Note:*** It is not required to remove the total amount of volume for all added reagents combined (1.75 mL) from the stock Digest Solution when preparing the Complete Digest Solution.

**Table:** Awesome wash buffer

Reagent	Final concentration	Amount
diH_2_O	N/A	(Up to) 846 mL
BSA	N/A	5 g
Dextrose (D-glucose)	N/A	4.5 g
EDTA (0.5 M)	2 mM	4 mL
PBS (10×)	1×	100 mL
L-glutamine (200 mM)	2 mM	10 mL
Sodium pyruvate (100 mM)	1 mM	10 mL
Non-essential Amino-Acids (100×)	1×	10 mL
Essential Amino-Acids (50×)	1×	20 mL
**Total**	**N/A**	**1 L**

Store sterile buffer at 2°C–8°C for up to one month.

**CRITICAL:** Check and adjust the pH of the Awesome Wash Buffer to 7.4.

**CRITICAL:** Sterile filter the Awesome Wash Buffer through a 0.22 μm polyethersulfone (PES) vacuum filter.

***Note:*** After sterile filtering, the buffer can be stored at 2°C–8°C for up to one month, if the bottle remains unopened or is opened only in a sterile laminar flow hood.

**Table:** Red blood cell lysis buffer

Reagent	Final concentration	Amount
Sterile deionized water	N/A	45 mL
Red blood cell lysis buffer (10×)	1×	5 mL
**Total**	**N/A**	**50 mL**

Store buffer at 2°C–8°C or on ice for up to 24 h.

***Note:*** The Red blood cell lysis buffer is prepared from the Biolegend 10× Buffer.

**Table:** Staining buffer

Reagent	Final concentration	Amount
Awesome wash buffer	N/A	8 mL
Brilliant stain buffer (5×)	1×	2 mL
**Total**	**N/A**	**10 mL**

Store buffer at 2°C–8°C or on ice for up to 24 h.

***Note:*** The Staining buffer is prepared from the Brilliant Stain buffer (Thermo Fisher Scientific).

**Table:** Fix/Perm buffer

Reagent	Final concentration	Amount
Diluent	N/A	7.5 mL
Concentrate (4×)	1×	2.5 mL
**Total**	**N/A**	**10 mL**

Store at 2°C–8°C for up to 24 h.

***Note:*** The Fix/Perm buffer is prepared from the eBioscience Foxp3/transcription factor Staining Buffer set.

**Table:** Perm buffer

Reagent	Final concentration	Amount
Deionized water	N/A	45 mL
Perm buffer (10×)	1×	5 mL
**Total**	**N/A**	**50 mL**

Store at 2°C–8°C for up to 24 h.

***Note:*** The Perm buffer is prepared from the eBioscience Foxp3/transcription factor Staining Buffer set.

## Step-by-step method details

### Preparation of materials

**Timing: 30 min**

In this section, we will detail the steps to prepare and label materials for tissue collection.

1. Label a 15 mL tube for each tissue to be collected.
2. Label a 50 mL tube for each tumor and spleen tissue to be collected.
3. Label 12-well plates; label one well for each lymph node, spleen and tumor to be collected.
4. Label 2 weigh boats for each tumor and spleen tissue to be collected.

***Optional:*** If the CD45+ and CD31+ enrichment steps are performed, label 4 ∗ 15 mL tubes for each tumor to be collected (CD45-positive, CD45-negative, CD31-positive and CD31-negative).

### Preparation of harvest solutions

**Timing: 30 min**

In this section, we outline the preparation of harvest solutions for tissue processing.

5. Prepare the 500 mL bottle of Stock Digest Solution (everything except DNase I and Liberase), according to the Materials setup section.
6. Prepare the Awesome Wash Buffer according to the Materials setup section.

### Preparation of extracellular antibody cocktails and solutions

**Timing: 2–3 h**

This section describes the preparation of extracellular antibody cocktails and solutions essential for staining various cell types.

**CRITICAL:** NK cell markers are strain specific. In BALB/cAnNRj mice the NK cell marker expressed is NKp46. In C57Bl/6J mice the expressed NK cell marker is instead NK1.1. The antibody used in the panels in this protocol detects NKp46. Please ensure usage of an antibody that detects NK cells in the specific strain of mice used in the experiment.

7. To prepare the extracellular antibody cocktails: Label tubes, one tube for every extracellular panel.
  a. Tumor infiltrating lymphocytes (TILs) – one tube.
  b. Tumor infiltrating myeloid cells (TIMs) – one tube.
  c. Spleen/LN lymphocytes – one tube.
  d. Spleen/LN myeloid cells – one tube.
8. To prepare the FMO control cocktails: Label each tube with the name of the extracellular marker fluorophore that will be missing in the solution and tissue type. Label one set of FMO tubes per panel and tissue type.

**Table 1:** Antibody cocktail for extracellular staining of tumor infiltrating lymphocytes

Marker/Reagent	Fluorophore	Clone	Final dilution	μL/sample (total 50 μL)
Staining Buffer	N/A	N/A	N/A	46.71
CD11b	BV510 (dump)	M1/70	1:400	0.125
CD45	BV786	30-F11	1:300	0.167
CD3	PerCPCy5.5	145-2C11	1:50	1
B220	PE	RA3-6B2	1:100	0.5
NKp46	FITC	29A1.4	1:200	0.25
CD4	BV605	RM4-5	1:200	0.25
CD8	APC	53–6.7	1:200	0.25
CD25	PECy7	PC61	1:200	0.25
CD44	BV421	IM7	1:200	0.25
CD62L	BV711	Mel-14	1:200	0.25

Store at 2°C–8°C for up to 24 h.

***Note:*** As an example, for the tumor infiltrating lymphocyte panel, label one tube each (10 tubes in total) with all the fluorophores listed in the extracellular tumor infiltrating lymphocyte panel (Table 1): E.g. “BV510 TIL”, “BV786 TIL” etc. (See example in supplemental information Figure S1).

9. For the cell-based compensation control, label one tube with “BV510 comp”.
10. Calculate the volume of staining buffer that needs to be prepared per panel.

***Note:*** The volume needed for the tumor lymphocyte panel and for the tumor myeloid panel: 50 μL ∗ (number of samples + Intracellular marker FMO + extra for pipetting).

Example: If 10 tumors will be harvested:

50 μL ∗ (10 tumors + 1 Intracellular marker FMO + 3 extra for pipetting).

= 50 μL ∗ 14.

= 700 μL.

The volume needed for the spleen/LN lymphocyte panel: 50 μL ∗ ((2 ∗ number of samples) + (1 ∗ Intracellular marker FMO) + extra for pipetting).

Example: If 10 tumor draining lymph nodes and 10 spleens will be harvested:

50 μL ∗ ((2 ∗ 10 samples) + (1 Intracellular marker FMO) + 4 extra for pipetting).

= 50 μL ∗ 25.

= 1250 μL.

The volume needed for the spleen/LN myeloid panel: 50 μL ∗ ((2 ∗ number of samples) + extra for pipetting).

Example: If 10 tumor draining lymph nodes and 10 spleens will be harvested:

50 μL ∗ ((2 ∗ 10 samples) + 4 extra for pipetting).

= 50 μL ∗ 24.

= 1200 μL.

***Note:*** For all panels combined there are 55 extracellular marker FMO samples (one set of FMO controls per panel and tissue) for which you need to prepare 200 μL each.

(55 FMO’s + 2 extra for pipetting) ∗ 200 μL = 11400 μL.

***Note:*** Two cell-based compensation controls need to be prepared, the unstained control and the control stained with the L/D dye and dump antibodies. For these 200 μL each will be used.

200 μL ∗ (2 + 1 extra for pipetting) = 600 μL.

***Note:*** Calculate the total volume of Staining Buffer to be prepared.

According to the examples used above, with 10 animals, this would equal:

700 μL + 700 μL + 1250 μL + 1200 μL + 11400 μL + 600 μL = 15850 μL.

11. To prepare Staining Buffer combine 4 parts of Awesome Wash Buffer with 1 part Brilliant Stain Buffer (Thermo Fisher Scientific).

***Note:*** For the total of 15850 μL Staining Buffer (as in the example in step 10), combine 12680 μL Awesome Wash Buffer with 3170 μL Brilliant Stain Buffer.

***Note:*** Brilliant Stain Buffer reduces non-specific interactions between the Brilliant fluorophores (i.e. BV605 and BV510). It is therefore recommended to use in the antibody cocktail if you have two or more of the Brilliant fluorophores.

12. Calculate the volume of each antibody to be added to the Staining Buffer, according to Tables 1, 2, 3, and 4.

**Table 2:** Antibody cocktail for extracellular staining of tumor infiltrating myeloid cells

Marker/Reagent	Fluorophore	Clone	Final dilution	μL/sample (total 50 μL)
Staining Buffer	N/A	N/A	N/A	47.21
CD19	BV510 (dump)	6D5	1:200	0.25
TCRβ	BV510 (dump)	H57-597	1:200	0.25
NKp46	BV510 (dump)	29A1.4	1:200	0.25
CD45	BV786	30-F11	1:300	0.167
CD11b	BV711	M1/70	1:400	0.125
Ly6G	BV605	1A8	1:200	0.25
Ly6C	APC/Fire750	HK1.4	1:200	0.25
CD11c	PECy7	HL3	1:200	0.25
I-A/I-E MHC II	BV421	M5/114.15.2	1:200	0.25
F4/80	PerCPCy5.5	BM8	1:100	0.5
CD86	PE	GL-1	1:200	0.25

Store at 2°C–8°C for up to 24 h.

**Table 3:** Antibody cocktail for extracellular staining of lymphocytes in secondary lymphoid tissue

Marker/Reagent	Fluorophore	Clone	Final dilution	μL/sample (total 50 μL)
Staining Buffer	N/A	N/A	N/A	46.96
CD11b	BV510 (dump)	M1/70	1:400	0.125
CD45	BV786	30-F11	1:300	0.167
CD3	PerCPCy5.5	145-2C11	1:50	1
B220	PE	RA3-6B2	1:100	0.5
NKp46	FITC	29A1.4	1:200	0.25
CD4	BV605	RM4-5	1:200	0.25
CD8	APC	53–6.7	1:200	0.25
CD25	PECy7	PC61	1:200	0.25
PD1	BV421	29F.1A12	1:200	0.25

Store at 2°C–8°C for up to 24 h.

**Table 4:** Antibody cocktail for extracellular staining of myeloid cells in secondary lymphoid tissue

Marker/Reagent	Fluorophore	Clone	Final dilution	μL/sample (total 50 μL)
Staining Buffer	N/A	N/A	N/A	46.46
CD19	BV510 (dump)	6D5	1:200	0.25
TCRβ	BV510 (dump)	H57-597	1:200	0.25
NKp46	BV510 (dump)	29A1.4	1:200	0.25
CD45	BV786	30-F11	1:300	0.167
CD11b	BV711	M1/70	1:400	0.125
Ly6G	BV605	1A8	1:200	0.25
Ly6C	APC/Fire750	HK1.4	1:200	0.25
CD8a	Alexa 488	53–6.7	1:200	0.5
CD11c	PECy7	HL3	1:200	0.25
I-A/I-E MHC II	BV421	M5/114.15.2	1:200	0.25
Siglec H	PE	551	1:100	0.5

Store at 2°C–8°C for up to 24 h.

***Note:*** If 10 tumors will we harvested according to the example in step 10, a final dilution of 1:200 of an antibody in the tumor infiltrating lymphocyte cocktail would mean that 700 μL/200 = 3.5 μL antibody should be added to 700 μL Staining Buffer.

13. Add the calculated volume of Staining Buffer minus the calculated volume of all the antibodies to be added to each labeled tube (Tables 1, 2, 3, and 4).
14. For each panel; add all the extracellular antibodies according to Tables 1, 2, 3, and 4, to the labeled tubes with Staining Buffer.
15. To prepare the FMO control cocktails: Calculate how much Staining Buffer and antibody to add to each tube. Adjust the volume to use a minimum of 0.5 μL of each antibody in the FMO cocktails (supplemental information Figure S1).
  ***Note:*** As an example, for the tumor infiltrating lymphocyte panel, the largest dilution is 1:400 for CD11b.
  Add 200 μL Staining Buffer to the FMO tubes of this panel.
  Add 200/400 = 0.5 μL of the antibody with the 1:400 dilution (CD11b) to all tubes except the BV510 TIL FMO tube.
  Add 200/300 = 0.67 μL of the antibody with the 1:300 dilution (CD45) to all tubes except the BV786 TIL FMO tube.
  Add 200/200 = 1 μL of the antibodies with the 1:200 dilution:
  NKp46 to all tubes except the FITC TIL FMO tube.
  CD4 to all tubes except the BV605 TIL FMO tube.
  CD8 to all tubes except the APC TIL FMO tube.
  CD25 to all tubes except the PECy7 TIL FMO tube.
  CD44 to all tubes except the BV421 TIL FMO tube.
  CD62L to all tubes except the BV711 TIL FMO tube.
  Add 200/100 = 2 μL of the antibody with the 1:100 dilution (B220) to all tubes except the PE TIL FMO tube.
  Add 200/50 = 4 μL of the antibody with the 1:50 dilution (CD3) to all tubes except the PerCPCy5.5 TIL FMO tube.

16. To prepare the cell-based compensation control antibody solution: Label one tube with BV510 comp.
  a. Add 200 μL Staining Buffer to the tube.
  b. Add all dump channel antibodies (from all panels used) to the tube, according to their respective dilutions:
    i. 0.5 μL CD11b, 1 μL CD19, 1 μL TCRβ and 1 μL NKp46.
  c. Save the rest of the Staining Buffer for the unstained control.
17. Store the antibody cocktails, FMO control cocktails and cell-based compensation control solution in the dark at 2°C–8°C until staining can be initiated. The cocktails should be used within 24 h.

### Final preparation of harvest solutions

**Timing: 30 min**

This section details the essential steps for preparing the solutions and buffers needed for tissue harvest.

18. Place prelabeled tubes, plates and weigh boats on ice to precool.
19. Prepare Complete Digest Solution by adding Liberase and DNase I to the appropriate amount of Stock Digest Solution as outlined in the materials and equipment section:
  a. Prepare 5 mL per tumor, 2 mL per spleen and 1 mL per set of lymph nodes of Complete Digest Solution.

***Note:*** Example calculation for 10 animals:

Stock Digest Solution: 10 ∗ 5 mL (tumor) + 10 ∗ 2 mL (spleen) + 10 ∗ 1 mL (lymph nodes) + 5 mL for pipetting.

= 85 mL.

Add 85 ∗ 7.5 μL Liberase.

= 637.5 μL.

Add 85 ∗ 10 μL DNase I.

= 850 μL.

**CRITICAL:** Complete Digest Solution should always be prepared fresh from the Stock Digest Solution, stored at 2°C–8°C or on ice and used within 24 h.

20. Add Complete Digest Solution to the pre-cooled 12-well plate for lymph nodes, 1 mL per well.
21. Add Awesome Wash Buffer to the 12-well plates for spleen and tumor, 1 mL per well.

***Note:*** Lymph nodes can be placed directly into Complete Digest Solution, and kept on ice for up to one hour. If it is expected to take longer than one hour, it is advisable to initially transfer them to Awesome Wash Buffer and then move them to a second plate containing Complete Digest Solution before mechanical dissociation.

22. Dilute Red Blood Cell (RBC) Lysis Buffer 1:10 in sterile deionized water.

***Note:*** Example calculation for 10 animals:

Total amount of RBC lysis buffer to prepare for all tissues: 10 ∗ 2 mL (tumor) + 10 ∗ 2 mL (spleen) + 10 ∗ 2 mL (lymph nodes) + 1 ∗ 2 mL (extra, for pipetting).

= 62 mL.

Prepare a bottle with 0.9 ∗ 62 mL deionized water.

= 55.8 mL.

Add 0.1 ∗ 62 mL 10× RBC lysis buffer.

= 6.2 mL.

23. Pre-warm RBC Lysis Buffer to 37°C.
24. Pre-cool the centrifuge to 4°C.

### Tissue harvest

**Timing: 0.5–2 h, depending on sample size**

This section outlines the steps for efficiently harvesting tissues, ensuring optimal sample integrity and preparation for subsequent processing.

25. Collect tumors and spleens, place on a precooled weigh boat and keep them on ice.
26. Weigh each tumor and spleen on a sensitive scale (in milligrams).
27. Place tumors and spleens in Awesome Wash Buffer, in 12-well plates on ice.
28. Harvest lymph nodes and place in 1 mL Complete Digest Solution, in 12-well plates on ice.

***Note:*** Multiple lymph nodes can be combined in one well.

**CRITICAL:** Work quickly and keep samples on ice continuously as you harvest all tissues.

***Note:*** Tumors and spleens are to be weighed before placed in Awesome Wash Buffer. This will provide data on number of a specific immune cell population per mg of tumor or spleen.

**Figure 1:**
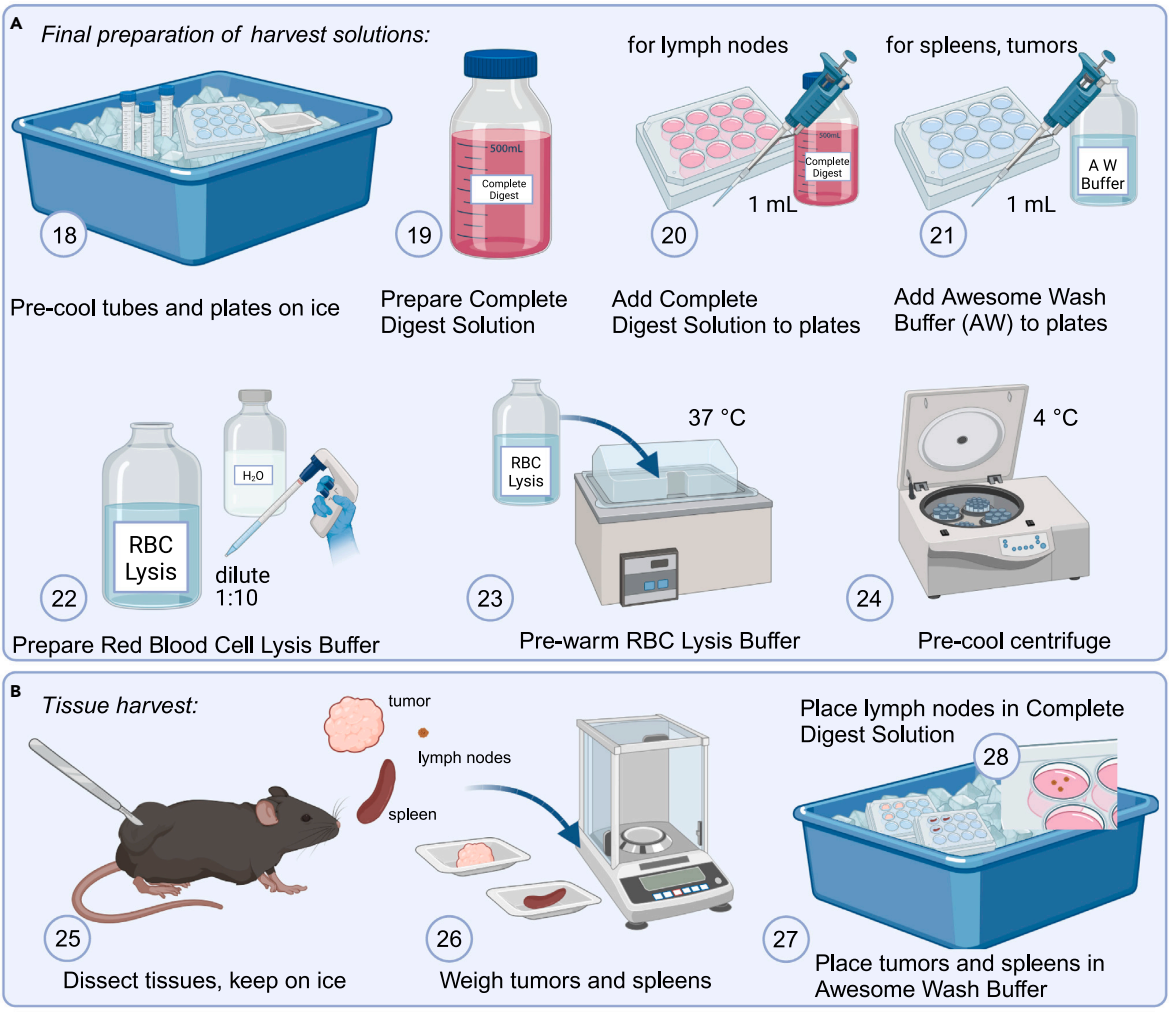
Schematic overview of preparation and tissue harvest, related to steps 18–28 (A) Initial preparation steps including buffer preparation of Complete Digest Solution, Awesome Wash Buffer and Red Blood Cell (RBC) Lysis Buffer as well as pre-cooling/warming of solutions and materials. (B) Harvest of tumor, spleen and lymph nodes. Weighing of the tumors and spleens and transfer to 12-well plates on ice. Several lymph nodes can be combined in one well.

***Note:***Figure 1 illustrates the preparation of harvest solutions (Figure 1A) and the tissue harvest steps (Figure 1B).

### Tissue processing and digestion

**Timing: 2–4 h, depending on sample size**

This section describes the procedure for processing and digesting various tissues to prepare them for further analysis.

29. For tumors:
  a. Take the tissue from the Awesome Wash Buffer and place on a weigh boat.
  b. Mince tissue with sharp scissors until pieces are approximately 0.5 mm in length/depth.
  c. Immediately after mincing, take up 5 mL of Complete Digest Solution and transfer tissue pieces from the weigh boat into the labeled 15 mL tube.
  d. Rinse the weigh boat thoroughly into the tube to collect all pieces.
  e. Place the tube back on ice until all tumors are minced.
  f. Choose one of the following digestion methods, once all tumors are processed:
    i. Transfer them to a 37°C water bath and incubate for 15 min.
    ii. Or: Transfer them to a 37°C incubator and incubate for 30 min.
  g. While incubating, pipet up and down with a 1 mL pipet every 5–10 min (at least 3 times):
    i. Use either pipet tips with approximately 2 mm cut off, or wide orifice pipet tips, to reduce blockage by tissue pieces.
    ii. Pipet approximately 20× per tumor.

***Note:*** There is no strict size limit for the tumors; however, for optimal results, they should generally range between 50 mm³ and 1,500 mm³. Processing time may vary depending on the tumor type and size. In general, larger, collagen dense tumors may need additional time and reagents for proper dissociation, while smaller, less dense tumors tend to dissociate more quickly. However, longer incubation increases the risk of cleaving certain markers, such as CD8. Therefore, the incubation time should be optimized to find a balance that allows proper tumor digestion while preserving marker integrity for flow cytometry evaluation.

30. For spleens:
  a. Take the tissue from the Awesome Wash Buffer and place on a weigh boat.
  b. Mince tissue with sharp scissors until pieces are approximately 0.5 mm in length/depth.
  c. Immediately after mincing, take up 2 mL of Complete Digest Solution and transfer tissue pieces from the weigh boat into the labeled 15 mL tube.
  d. Rinse the weigh boat thoroughly into the tube to collect all pieces.
  e. Place the tube back on ice until all spleen tissues are processed.
  f. Choose one of the following digestion methods, once all spleens are processed:
    i. Transfer them to a 37°C water bath and incubate for 15 min.
    ii. Or: Transfer them to a 37°C incubator and incubate for 30 min.
  g. While incubating, pipet up and down with a 1 mL pipet every 5–10 min (at least 3 times):
    i. If necessary, use pipet tips with approximately 2 mm cut off, or wide orifice pipet tips.
    ii. Pipet approximately 20× per tissue.
31. For lymph nodes, see also^8^:
  a. If lymph nodes are in Awesome Wash Buffer, transfer them to a new 12-well plate with 1 mL Complete Digest Solution.
  b. Use two 26G syringe needles to tear apart the lymph nodes within the well of the 12-well plate. Ensure to break the lymph node capsule completely.
  c. Choose one of the following digestion methods, once all lymph nodes are processed:
    i. Transfer the plate with lymph nodes to a 37°C incubator and incubate for 30 min.
    ii. Or: Transfer the solution including lymph node pieces to a prelabeled 15 mL tube and transfer the tubes to a 37°C water bath and incubate for 15 min.
  d. While incubating, pipet up and down with a 1 mL pipet every 5–10 min (at least 3 times):
    i. If necessary, use pipet tips with approximately 2 mm cut off, or wide orifice pipet tips.
    ii. Pipet approximately 20× per tissue.
32. Incubate all tissues for an additional 10 min on ice.
33. Homogenize tissues through a filter:
  a. Apply solution including all tissue bits to a 70 μm filter on top of a clean 50 mL tube.
  b. Smash the tissue bits with the plunger of a syringe.
  c. Rinse the syringe and the filter with 10 mL Awesome Wash Buffer to ensure full cell recovery.
34. Centrifuge for 5 min at 350 × *g*, at 4°C.
35. Discard the supernatant.
36. Resuspend the pellet in 2 mL RBC Lysis Buffer.
37. Incubate for 4 min at 37°C in a water bath or for 5 min at 18°C–25°C.
38. Add 10 mL Awesome Wash Buffer to dilute.
39. Proceed to separation or directly to staining procedure.

**Figure 2:**
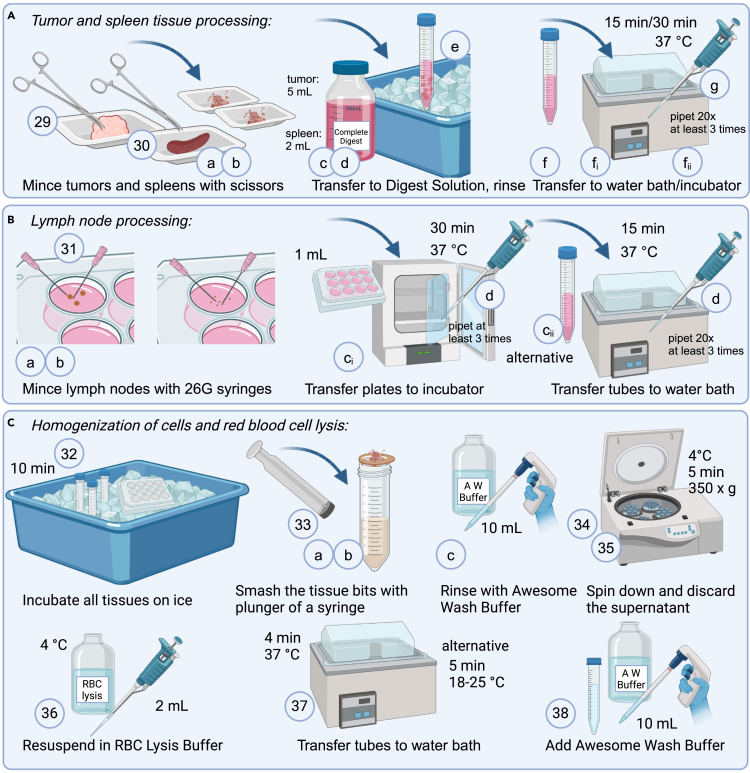
Schematic overview of tissue processing and digestion, related to steps 29–38 (A) Tumor tissue and spleen tissue processing, (B) Lymph node processing and (C) the homogenization of cells and red blood cell lysis.

***Note:***Figure 2 illustrates the tissue processing and digestion steps for tumors, spleens (Figure 2A), lymph nodes (Figure 2B) and the homogenization of cells and Red Blood Cell lysis (Figure 2C).

### Separation of CD45-positive immune cells

**Timing: 1–2 h, depending on sample size and number of available slots in magnetic stand/AutoMACS**

This section describes the process for isolating of CD45-positive immune cells using magnetic-activated cell sorting (MACS), as an optional step in the protocol.

***Note:*** CD45 enrichment ensures staining of a clean immune cell population for flow cytometry. This can be particularly important in tumor models with few immune cells relative to tumor and/or other stromal cells.

***Note:*** In this protocol, the MACS Miltenyi magnetic bead selection is used. However, other bead-based enrichment kits could be used as well to replace these steps.

40. Prepare CD45 enrichment bead solution by adding 50 μL of mouse CD45 MicroBeads to 450 μL Awesome Wash Buffer.
  ***Note:*** Example calculation for 10 tissues:
  450 μL ∗ (10 tumors + 1 extra for pipetting) Awesome Wash Buffer.
  = 4950 μL.
  Add 50 μL ∗ (10 tumors + 1 extra for pipetting) mouse CD45 MicroBeads.
  = 550 μL.
41. Add 1:400 Fc block (2.4G2) to the CD45 enrichment bead solution.
  ***Note:*** Example calculation for 10 tissues.
  = 5500 μL enrichment bead solution.
  Add 1:400 Fc block.
  = 5500 μL / 400.
  = 13.75 μL.
42. Spin samples for 5 min at 350 × *g*, at 4°C.
43. Discard the supernatant.
44. Resuspend the cell pellet from each sample in 500 μL CD45 enrichment bead solution.
45. Incubate for 15 min, at 2°C–8°C.
46. Add 5 mL Awesome Wash Buffer to the tube to dilute the sample.
47. Transfer the whole sample to a 70 μM filter on top of a fresh 15 mL conical tube.
48. Rinse the filter with an additional 5 mL Awesome Wash Buffer to ensure full cell recovery.
49. Spin samples for 5 min at 350 × *g*, at 4°C.
50. Discard the supernatant.
51. Resuspend the cells in 2 mL Awesome Wash Buffer.
52. Follow the enrichment steps from the mouse CD45 MicroBeads protocol, either on an AutoMACS instrument or with LS columns in a magnetic stand.
53. Take the CD45-positive fraction and proceed to the immune cell staining protocol; and/or, take the CD45-negative fraction to proceed to the CD31+ enrichment protocol.

### MACS separation of CD31-positive endothelial cells

**Timing: 1–2 h, depending on sample size and number of available slots in magnetic stand/AutoMACS**

This section describes the process for isolating CD31-positive endothelial cells using magnetic-activated cell sorting (MACS), as an optional step in the protocol.

***Note:*** Most tumor models contain a relatively low fraction of endothelial cells, thus, this enrichment step is generally critical for obtaining a sufficient number of cells for effective staining and analysis.

54. Prepare CD31 enrichment bead solution by adding 50 μL of mouse CD31 MicroBeads to 450 μL Awesome Wash Buffer for each sample.
  ***Note:*** Example calculation for 10 tissues:
  450 μL ∗ (10 tumors + 1 extra for pipetting) Awesome Wash Buffer.
  = 4950 μL.
  Add 50 μL ∗ (10 tumors + 1 extra for pipetting) mouse CD31 MicroBeads.
  = 550 μL.
55. Add 1:400 Fc block (2.4G2) to the CD31 enrichment bead solution.
  ***Note:*** Example calculation for 10 tissues.
  = 5500 μL enrichment bead solution.
  Add 1:400 Fc block.
  = 5500 μL / 400.
  = 13.75 μL.
56. Spin samples for 5 min at 350 × *g*, at 4°C.
57. Discard the supernatant.
58. Resuspend the cell pellet in 500 μL CD31 enrichment bead solution.
59. Incubate for 15 min, at 2°C–8°C.
60. Add 5 mL Awesome Wash Buffer in the tube to dilute the sample.
61. Transfer the whole sample to a 70 μm filter on top of a prelabeled 50 mL conical tube.
62. Rinse the filter with an additional 5 mL Awesome Wash Buffer to ensure full cell recovery.
63. Spin samples for 5 min at 350 × *g*, at 4°C.
64. Discard the supernatant.
65. Resuspend the cells in 2 mL Awesome Wash Buffer.
66. Follow the enrichment steps from the mouse CD31 MicroBeads protocol, either on an AutoMACS instrument or with LS columns in a magnetic stand.
67. Proceed to staining protocol for endothelial cells.

***Note:*** The CD31 enrichment and staining of endothelial cells have not been performed for this manuscript and therefore this is not described further in the staining and analysis sections. However, the protocol was developed to ensure optimal immune and endothelial cell viability, as previously described,^9^ and can therefore be used for this purpose.

### Antibody staining of samples

**Timing: 2.5–20 h**

This section outlines the procedure for staining the isolated cells with antibodies, allowing for the identification of specific cell populations via flow cytometry.

68. Centrifuge samples at 350 × *g* for 5 min at 4°C.
69. Discard the supernatant.
70. Resuspend the cell pellet in the appropriate amount of Awesome Wash Buffer:
  a. Tumor samples after CD45 enrichment: 400–600 μL.
  b. Tumor samples without CD45 enrichment: 400 μL - 4 mL (depending on tumor size).
  c. Spleen samples: 1–4 mL.
  d. Lymph node samples: 400–600 μL.

***Note:*** For tumor samples that are analyzed without enriching for CD45+ immune cells, it is recommended to count the cells and not stain more than 5 × 10^7^ cells per sample.

71. Transfer 200 μL of each sample to the wells of a V- bottomed 96-well plate.
72. Prepare separate 96-well plates with cells for FMO controls and live/dead/BV510 single stain and unstained compensation control. See supplemental information Figure S2 for an example.

***Note:*** For the FMOs, live/dead single stain and unstained controls, leftover cells from multiple samples can be combined and distributed among wells to ensure enough cells can be analyzed in each control.

73. During the centrifugation and washing steps (steps 74–77 below), prepare Fc block and Live/Dead solutions for the samples, for the BV510 compensation control and for FMO controls (supplemental information Figure S2):
  a. Label one tube “Fc block” and label a second tube “Fc block + Live/Dead”.
  b. Calculate how much of the mixture that needs to be prepared for 50 μL/sample final volume. Add some extra volume for pipetting.
    ***Note:*** Example for Fc block + Live/Dead; if tissues were harvested from 10 mice: For 30 samples and 53 FMOs ((50 ∗ (30 + 53) + 350 extra) = 4500 μL PBS is needed.
    ***Note:*** Example for Fc block: For 6 FMOs and 1 unstained compensation control ((50 ∗ (6 + 1) + 150 extra) = 500 μL PBS is needed.
  c. Add the calculated volume of PBS into the pre-labeled tubes.
  d. Add Fc block and Live/Dead, both at a 1:400 dilution. Add only Fc block to the Fc block tube.
    ***Note:*** Example for 30 samples + 53 FMOs and a PBS volume of 4500 μL, add 4500/400 = 11.25 μL Fc block and add 4500/400 = 11.25 μL Live/Dead
    ***Note:*** Example for 6 FMOs + 1 unstained compensation control and a PBS volume of 500 μL, add 500/400 = 1.25 μL Fc block
  e. Mix by pipetting up and down a few times.
74. Centrifuge the 96-well plates at 350 × *g* for 5 min at 4°C.
75. Discard the supernatant by flicking off the supernatant in one firm movement.
76. Place the plates on ice.
77. Wash all wells with 1× PBS (2×):
  a. Resuspend all samples and controls in 200 μL 1× PBS.
  b. Centrifuge the 96-well plates at 350 × *g* for 5 min at 4°C.
  c. Discard the supernatant by flicking off the supernatant in one firm movement.
78. Add the prepared Fc block and Live/Dead solutions to the samples:
  a. Transfer the solutions to two separate and labeled reservoirs.
  b. To the 96-well plate with cell pellets, use a multichannel pipette to add 50 μL/sample of:
    i. Fc block + Live/Dead: to all samples, to the BV510 compensation control and to all FMO controls except the BV510 FMO (Example shown in supplemental information Figure S2).
    ii. Fc block: to the non-stained compensation control and to the BV510 FMO controls (Example shown in supplemental information Figure S2).
  c. Mix by pipetting up and down approximately 10 times.
  d. Place the lid on the 96-well plate and incubate in the dark at 18°C–25°C for 10 min.
79. Wash all wells directly after Fc block and Live/Dead staining:
  a. Add 150 μL ice cold Awesome Wash Buffer with a multichannel pipet.
  b. Centrifuge the plates at 350 × *g*, for 5 min, at 4°C.
  c. Flick off the supernatant in one firm movement and place the 96-well plate on ice.
80. For extracellular staining:
  a. Label reservoirs and transfer cocktails from the prepared extracellular cocktail tubes to the reservoirs.
  b. Add 50 μL per sample of: antibody cocktails to samples and FMO cocktails to FMO controls (supplemental information Figure S3).
  c. Add Staining Buffer to the unstained compensation control.
  d. Add the prepared BV510 antibody cocktail to the BV510 compensation control.
  e. Mix by pipetting up and down approximately 10 times.
  f. Incubate for 30 min on ice in the dark, with the lid placed on the 96-well plate.
    i. In the meanwhile, prepare the Fix/Perm buffer and Perm buffer:
    ii. To prepare Fix/Perm buffer, mix 1 part concentrate with 3 parts diluent. Store at 2°C–8°C or on ice, discard after 24 h.
      ***Note:*** Calculate how much Fix/Perm buffer you need to prepare: 100 μL ∗ (number of samples + number of FMO controls + number of compensation controls) + extra for pipetting.
      The number of samples = Number of mice ∗ (number of panels ∗ number of tissues).
      Example: If 10 mice are used in the experiment to investigate myeloid and lymphoid cells (2 separate panels) in tumors, tumor draining lymph nodes and spleens (3 different tissues), the number of samples will be: 10 ∗ (2 ∗ 3) = 60 samples.
      Number of controls = 59 FMO controls + 1 single stained control + 1 unstained control = 61 controls.
      Extra = 15% of approximate volume = 0.15 ∗ 100 μL ∗ (60 samples + 61 controls) = 1.815 mL.
      Calculation of total volume needed:
      100 μL ∗ (60 samples + 59 FMO controls + 1 single stained cell control + 1 unstained cell control) + 1.8 mL extra for pipetting.
      = 100 μL ∗ (121) + 1.8 mL = 13.9 mL (can be rounded up to 14 mL).
      Dilution:
      14 mL / 4 = 3.5 mL.
      3.5 mL ∗ 3 = 10.5 mL.
      Mix 3.5 mL concentrate with 10.5 mL diluent.
    iii. To prepare 1× Perm buffer, dilute 1 part 10× Perm Buffer with 9 parts deionized water. Store at 2°C–8°C or on ice, discard after 24 h.
      ***Note:*** Calculate how much Perm buffer you need to prepare: (350 μL ∗ (number of samples + number of FMO controls + number of compensation controls)) + 200 μL ∗ ((number of samples/2) + lymphoid FMO controls) + extra for pipetting.
      The number of samples = Number of mice ∗ (number of panels ∗ number of tissues).
      Example: If 10 mice are used in the experiment to investigate myeloid and lymphoid cells (2 separate panels) in tumors, tumor draining lymph nodes and spleens (3 different tissues), the number of samples will be: 10 ∗ (2 ∗ 3) = 60 samples.
      Number of controls = 59 FMO controls + 1 single stained control + 1 unstained control = 61 controls.
      Extra = 15% of approximate volume = 0.15 ∗ ((350 μL ∗ (60 samples + 61 controls)) + 200 μL ∗ ((60 samples/2) + 31 lymphoid FMO controls)) = 0.15 ∗ ((350 ∗ 121) + (200 ∗ 61)) = 8182.5 μL.
      Calculation of total volume needed:
      350 μL ∗ (60 samples + 59 FMO controls + 1 single stained cell control + 1 unstained cell control) + 200 μL ∗ ((60 samples/2) + 31 FMO controls) + 8.2 mL extra for pipetting.
      = 350 μL ∗ (121) + 200 μL ∗ (61) + 8.2 mL extra.
      = 42 350 μL + 12 200 + 8200 μL.
      = 62.75 mL (can be rounded up to 63 mL).
      Dilution:
      63 mL/10 = 6.3 mL.
      63 mL - 6.3 = 56.7 mL.
      Mix 6.3 mL 10× Perm Buffer with 56.7 mL deionized water.
  g. Wash by adding 150 μL ice cold Awesome Wash Buffer by multichannel pipetting.
  h. Centrifuge the samples at 350 × *g*, for 5 min, at 4°C.
  i. Flick off the supernatant in one firm movement.
81. Fix all wells:
  a. Add 100 μL/well of prepared 1× Fix/Perm Buffer to all samples, FMO controls and compensation controls.
  b. Mix thoroughly with a multichannel pipet.
  c. Place the lid on the 96-well plate.
  d. Incubate for 30 min on ice in the dark.
82. Wash all wells directly after incubation:
  a. Add 150 μL ice cold 1× Perm Buffer/well.
  b. Centrifuge the 96-well plate at 400 × *g*, for 5 min, at 4°C.
  c. Flick off the supernatant in one firm movement.

***Note:*** After fixation it may be appropriate to raise the speed of the centrifuge (from 350 × *g* to 400 × *g*).

**Pause point:** After fixing the cells it is possible to resuspend the cells in 100 μL Awesome Wash Buffer/well to store them at 2°C–8°C for 12–24 h. Before continuing, ensure to centrifuge off the Awesome Wash Buffer and permeabilize the cells by washing in 200 μL 1× Perm Buffer. Centrifuge at 400 × *g*, for 5 min, at 4°C. Flick off the supernatant in one firm movement.

83. For the intracellular staining:
  a. Add 50 μL/well of the intracellular antibody solutions prepared in 1× Perm Buffer (Tables 5 and 6).

**Table 5:** Antibody solution for intracellular staining of tumor infiltrating lymphocytes and lymphocytes in secondary lymphoid tissue

Marker	Fluorophore	Clone	Final dilution	μl/sample (total 50 μL)
1× Perm buffer	N/A	N/A	N/A	49.5
Foxp3-Biotin	N/A	FJK-16s	1:100	0.5

Use directly after preparation.

**Table 6:** Antibody solution for intracellular staining of tumor infiltrating myeloid cells

Marker	Fluorophore	Clone	Final dilution	μl/sample (total 50 μL)
1× Perm buffer	N/A	N/A	N/A	49.5
Arginase I	Alexa 488	AlexF5	1:100	0.5

Use directly after preparation.
  b. Add only 1× Perm Buffer to the compensation controls and to the FMO controls that should not be stained with the intracellular antibodies (supplemental information Figure S4).
  c. Incubate for 30 min on ice in the dark (with the lid placed on the 96-well plate).
84. Wash all wells directly after incubation:
  a. Add 150 μL ice cold 1× Perm Buffer, centrifuge at 400 × *g* for 5 min at 4°C.
  b. Flick off the supernatant in one firm movement.
85. For the lymphoid panels:
  a. Add 50 μL/well Streptavidin-APC/Fire750 in 1× Perm Buffer (Table 7) to all appropriate samples and to the FMO controls except the APC/Fire750 FMO control.

**Table 7:** Staining solution for intracellular Foxp3 staining of tumor infiltrating lymphocytes and lymphocytes in secondary lymphoid tissue

Reagent	Fluorophore	Final dilution	μl/sample (total 50 μL)
1× Perm buffer	N/A	N/A	49.5
Streptavidin	APC/Fire750	1:100	0.5
  b. Incubate for 30 min on ice in the dark (place the lid on the 96-well plate).
  c. Wash by adding 150 μL ice cold 1× Perm Buffer and centrifuge at 400 × *g* for 5 min at 4°C.
  d. Flick off the supernatant with one firm movement.
86. Resuspend all samples and controls in 200 μL ice cold Awesome Wash Buffer.
87. Put samples on ice or store at 2°C–8°C for a maximum of one week.
88. Prepare the bead-based compensation controls (see instructions below, steps 92–103).
89. Filter the samples, FMO controls and single stain compensation controls.
90. Record all samples and controls on a flow cytometer.

***Note:*** Wash the flow cytometer regularly in between samples and/or panels to prevent clogging of the machine and ensure optimal and clear signal in all samples throughout.

91. Analyze the data in the FlowJo software (see instructions below, steps 104–115).

### Preparation of bead-based single stain controls

**Timing: 30 min**

This section describes the procedure for preparing bead-based single stain controls essential for accurate flow cytometry analysis.

92. Label a well or polystyrene FACS tube for each bead-based single stain control.
93. Add 200 μL of Awesome Wash Buffer to each well or tube.

**CRITICAL:** UltraComp eBeads Plus Compensation beads are not compatible with Brilliant Stain Buffer, therefore always use Awesome Wash Buffer to prepare bead-based single stain controls.

94. Mix UltraComp eBeads Plus Compensation Beads vigorously by inverting at least 10 times or by pulse-vortexing.
95. Add a couple of drops of compensation beads to a 1.5 mL Eppendorf tube.
96. Pipet 20 μL of the beads directly into the Awesome Wash Buffer within each well or tube.
97. Add 0.5 μL antibody to the appropriate well or tube.

***Note:*** Example: for the PE single stain control in the tumor infiltrating lymphocyte panel, add 0.5 μL of B220 PE-conjugated antibody.

98. Mix well with a multichannel pipet or by pulse-vortexing.
99. Incubate for 15–30 min, at 2°C–8°C.
100. Spin down at 400 × *g* for 5 min.
101. Discard the supernatant.
102. Resuspend the beads in 200–400 μL Awesome Wash Buffer.
103. Keep the samples in the dark and on ice or at 2°C–8°C until the analysis on the flow cytometer.

***Note:*** It is recommended to use compensation beads for each antibody-fluorophore combination used in the panel. Compensation beads bind antibodies in a highly efficient manner and thus, in most cases, create a stronger positive signal than the antibody as it would bind to its marker on cells.

***Note:*** If the bead-based single stain controls are prepared on the same day as they are collected on the flow cytometer, steps 100 and 101 are optional.

### Generation and evaluation of the compensation matrix in FlowJo software

**Timing: 1–2 h**

This section outlines the key steps for generating and evaluating a compensation matrix in FlowJo software, to correct spectral overlap between fluorophores in flow cytometry.

**CRITICAL:** Generate a separate compensation matrix for each panel, using single stain controls for each individual fluorophore and/or dye that was used.

104. Load all single stained controls (beads), live/dead single stain and unstained controls (cells) into the “compensation” group.
105. Navigate to Tools and click “Compensation”.
106. Select all parameters that are included in the panel and click “choose selected parameters”.
107. In the compensation window, select the correct negative and positive peaks for each parameter.

**CRITICAL:** Select negative peaks for the beads and cells separately, since these have a different background autofluorescence.

108. Click “View Matrix” to calculate the compensation matrix.
109. Within the matrix window, go manually through each single stain control sample and check if the compensation calculation has compensated out all spillover to other channels correctly:
  a. Under “Preview Sample”:
    i. select one of the single stain control samples, for example “PE” in the first dropdown menu.
    ii. Select “Size” in the second dropdown menu.
    iii. Under “View”, select “All by…” and select the channel for the current single stain control, so “PE” in this example (Optional for a clearer view).
    iv. Uncheck “Overlay Uncompensated” (Optional for a clearer view).
  b. Look through the plots and check how the PE signal is picked up into all other channels that are included in the panel.
  c. If there is spillover or overcompensation into another channel:
    i. Edit the compensation matrix by increasing or decreasing the value that reflects the compensated fraction for PE into that channel.
    ii. Go back to the plots and check if the spill-over or overcompensation is gone. If not, increase or decrease the value in the compensation matrix further.
  d. If there is no spillover in any channels, continue to the next single stain control sample, until all have been checked.
110. Apply the (edited) compensation matrix to all samples.

**Figure 3:**
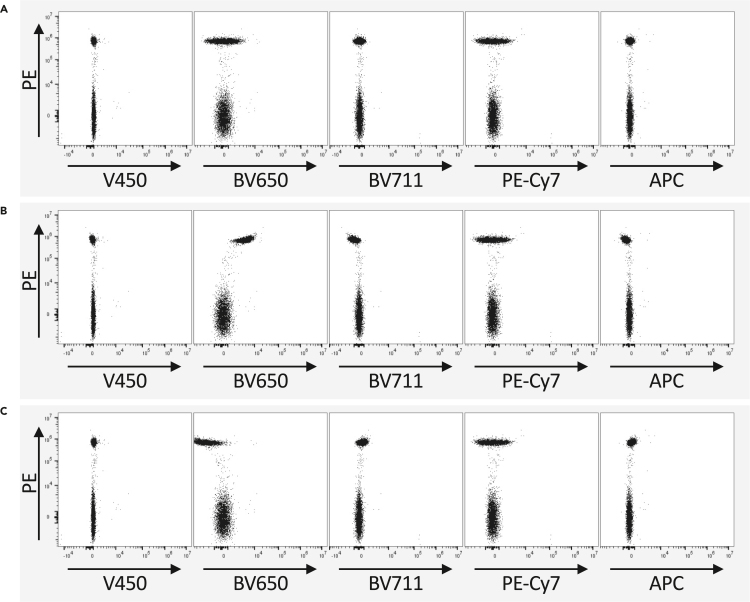
Compensation matrix check in FlowJo v.10 Example shown for the single stain control for PE in a panel with PE, V450, BV650, BV711, PE-Cy7 and APC. (A) Example of a properly calculated compensation matrix: PE does neither spill over into any channel, nor is it overcompensated in any channel. (B) Example of undercompensation: PE still spills over into the BV650 channel (named FL15-A::V660 on the flow cytometer used in this example). (C) Example of overcompensation: PE is compensated so much that it creates a negative signal in the BV650 channel.

***Note:*** For examples of spillover and overcompensation, see Figure 3.

### Gating of the different populations in FlowJo software

**Timing: 2–4 h per panel**

This section outlines the procedures for gating distinct cell populations in flow cytometry, ensuring accurate identification and analysis of immune cells.

**Figure 4:**
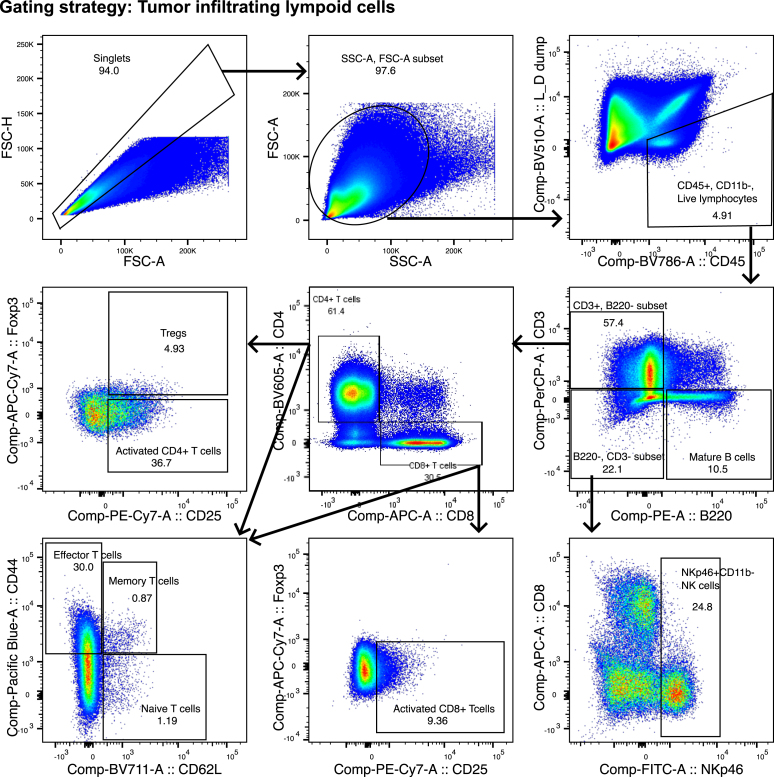
Gating strategy for the analysis of tumor infiltrating lymphocytes Select single cells based on size in the FSC-H vs. FSC-A gate. Exclude debris, the most granular cells and noise and include the cells of interest in the FSC-A vs. SCA-A gate. Select CD45+, CD11b-, Live cells, these are the CD11b-live lymphocytes. From the Live lymphocytes, gate on Mature B cells, the different T cell populations and Natural killer (NK) cells as indicated. Use the FMO controls to ensure correct gating. Figure reprinted and adapted with permission from Ingelshed et al. 2024^1^ (modifications applied).

**Figure 5:**
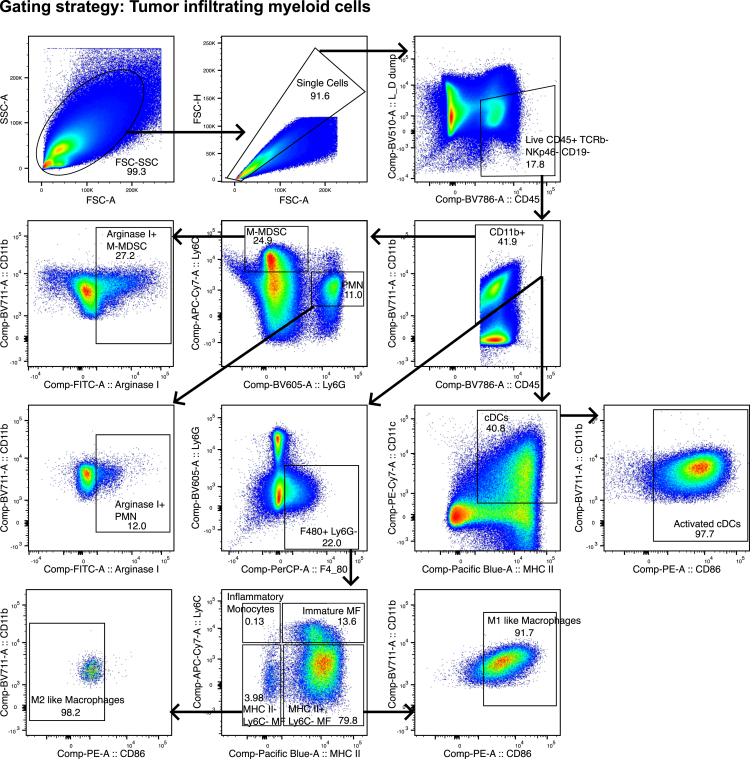
Gating strategy for the analysis of tumor infiltrating myeloid cells Select cells and exclude debris, noise and the most granular cells in the SSC-A vs. FSC-A gate. Select single cells, by excluding the largest cells/doublets in the FSC-H vs. FSC-A gate. Exclude T cells, B cells and NK cells and select live immune cells by gating on CD45+ Live/dead and dump channel negative cells. Choose the CD11b+ cells to select the myeloid cells. From live myeloid cells, gate as indicated on the Conventional Dendritic cell (cDC) subsets, Polymorphonuclear leukocyte (PMN) subsets, Monocytic-Myeloid derived suppressor cell (M-MDSC) subsets, Macrophage (MF) subsets and inflammatory monocytes. Use the FMO controls to ensure correct gating.

**Figure 6:**
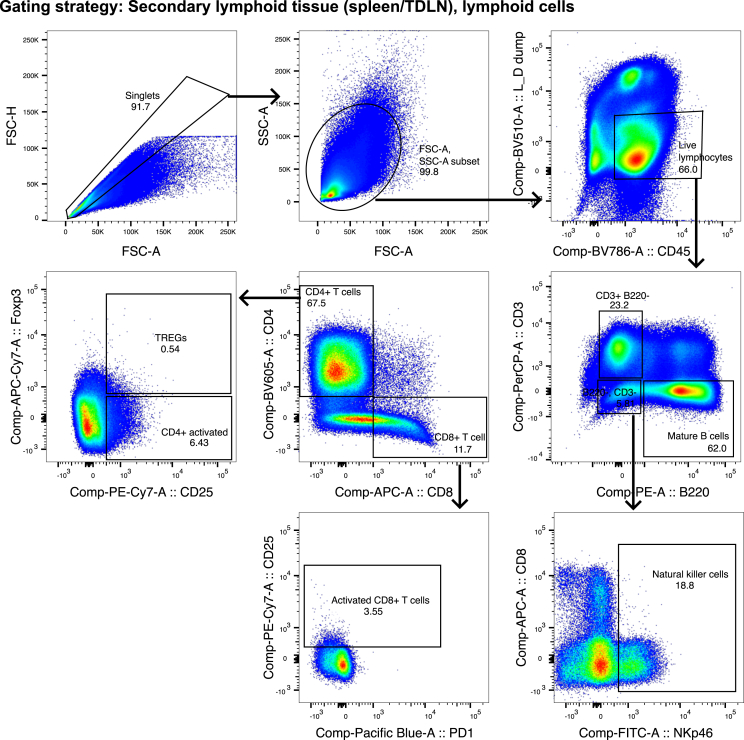
Gating strategy for the analysis of lymphocytes in secondary lymphoid tissue (spleen and lymph node) Select single cells based on size in the FSC-H vs. FSC-A gate. Exclude the most granular cells, debris and noise and include the cells of interest in the FSC-A vs. SCA-A gate. Select CD45+, dump-, Live/dead stain-, these are the CD11b-live lymphocytes. From the Live lymphocytes, gate on Mature B cells, the different T cell populations and Natural killer (NK) cells as indicated. Use the FMO controls to ensure correct gating.

**Figure 7:**
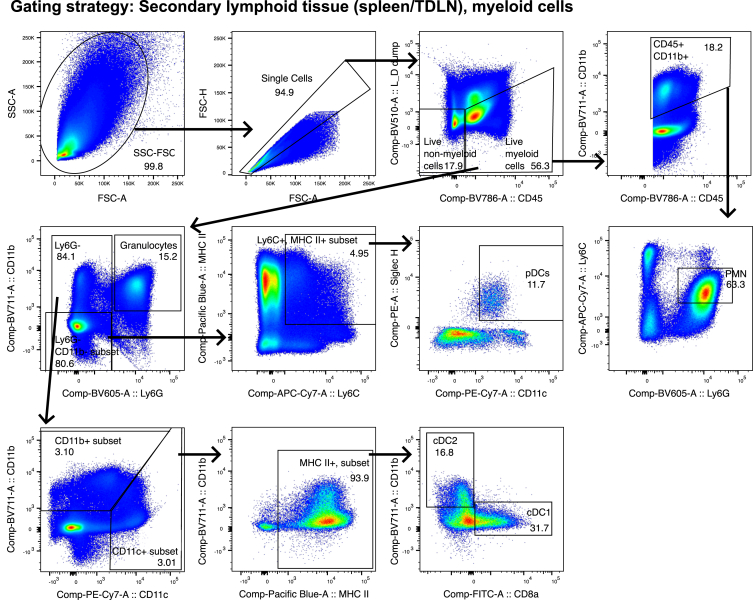
Gating strategy for the analysis of myeloid cells in secondary lymphoid tissue (spleen and lymph node) Select cells and exclude debris, noise and the most granular cells in the SSC-A vs. FSC-A gate. Select single cells, by excluding the largest cells/doublets in the FSC-H vs. FSC-A gate. Exclude T cells, B cells and NK cells and select live immune cells by gating on CD45+ Live/dead and dump channel negative cells. From these live myeloid cells, gate as indicated on the Polymorphonuclear leukocytes (PMNs), Plasmacytoid Dendritic cells (pDCs), and Conventional Dendritic cell (cDC) subsets. Use the FMO controls to ensure correct gating.

For all tissues and cell types (Figures 4, 5, 6, and 7).

111. Use forward scatter (FSC-A) and side scatter (SSC-A) to select cells and exclude debris.
112. Gate for single cells with FSC-Area versus FSC-Height.
113. Select fixable live-dead Aqua negative, CD45 positive cells to gate viable immune cells.

For specific tissues and staining panels.

**Figure 8:**
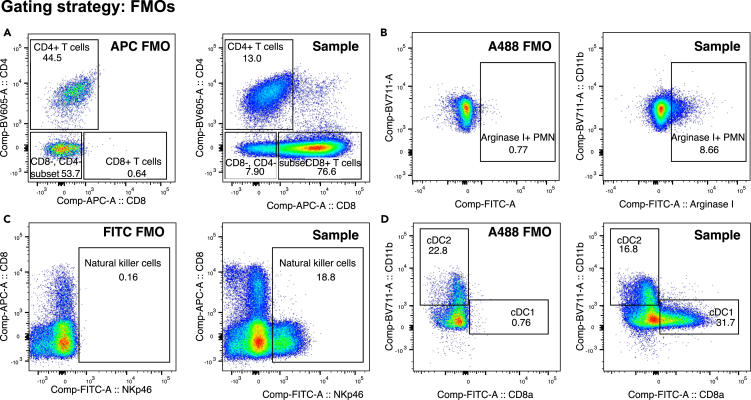
Examples of how to use FMO controls for gating A signal of < 1% in the FMO control is considered acceptable. (A) In the Tumor infiltrating lymphocyte panel (tumor tissue), the CD8+ CD4- population (CD8+ T cells) should be selected on the basis of where the population is lacking in the APC FMO control. Note that the CD8+ CD4+ population has a different position in regards to the negative APC signal in the FMO control than the CD8+ CD4- population has. (B) In the tumor infiltrating myeloid cell panel (tumor tissue), the Arginase I+ population can be selected depending on where the population is lacking in the FMO sample. (C) In the secondary lymphoid- spleen tissue- lymphocyte panel, to gate on the Natural killer cell population, include the population that is negative for the NKp46 marker in the FITC FMO control. (D) In the secondary lymphoid- spleen tissue- myeloid panel use the negative A488 population in the A488 FMO, to select type 1 Conventional Dendritic cells (cDC1).

***Note:*** Generally, a background signal of < 1% in the FMO control is considered acceptable.

***Note:*** An FMO control should be used for every marker for which a positive signal is unclear. i.e. CD8 expression on T cells is usually so clear that an FMO is not required to gate out CD8+ cells. However, it is always recommended to use FMO controls for all markers until this can be established for the specific marker within the specific staining panel and tissue type.

***Note:*** For antibody isotypes with a high background binding, for example for some intracellular markers, it is recommended to add the isotype control antibody to the FMO mix.

114. Follow the gating strategies as outlined in Figures 4, 5, 6, and 7 to gate for specific immune cell populations.
115. Use fluorescence minus one (FMO) controls for markers without a clear positive signal to ensure gates are set based on an appropriate level of background signal (Figure 8).

## Expected outcomes

Single-cell suspensions are obtained from tumor, spleen, and lymph node tissues, with minimal red blood cell contamination and debris. The protocol should yield viable cell populations ready for flow cytometric analysis, as indicated by low levels of dead cell staining. Flow cytometry staining of extensive immune cell marker panels will provide data on the expression of surface markers and intracellular markers, enabling differentiation between various immune cell types, such as T cells, B cells, and macrophages and their subpopulations. The obtained data will show proportions of these different immune cell types within tumor, spleen and lymph node tissues and allow for comparisons across different treatment conditions. Combined, it will reflect changes in the tumor microenvironment and systemic immune response.

## Quantification and statistical analysis

**Figure 9:**
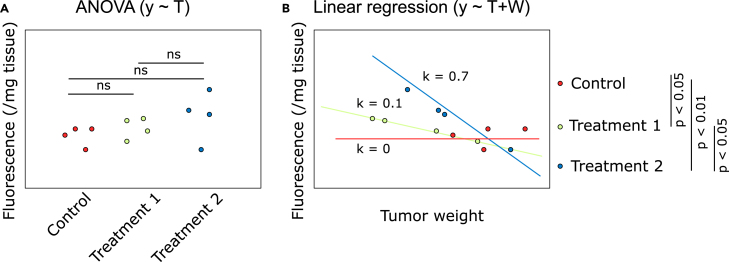
Comparison of data analysis methods using a fictive example with four tumors in each treatment group (*n* = 12) (A) Data analysis using ANOVA vs. (B) Data analysis using linear regression controlled by tumor weight. Given that treatment-induced tumor growth delay/shrinkage could lead to significant differences in tumor size at harvest, linear regression can be utilized to control for this potential confounder by including tumor size as a covariate.

There are several analytical methods that can be used to analyze the data from the experimental protocol above, and the choice of analytical method should be based on the research question which is investigated. In the following chapter, it is assumed that the effect of different anti-tumor treatments on immune cell infiltration is to be compared, that is the outcome (dependent) variable is the fluorescence signal and the predictor (independent) variables include the treatment regimens to be compared, as well as potential other factors of interest such as tumor weight at harvest. The most obvious method of analysis would be to perform ANOVA with a suitable post-hoc test such as Tukey’s test, and investigate whether there are significant differences observed between the treatments. However, the various treatments might lead to significantly different tumor size at harvest, which may be a confounding factor, based on the fact that a higher drug effect leads to tumor shrinkage. Thus, in this protocol we instead use multiple linear regression and include the tumor size in the regression as confounder control (Figure 9). Visually, it is clear that there is a trend in the data with a treatment weakly correlated with smaller tumors and increased infiltration (Treatment 1) and a treatment strongly correlated with smaller tumors and increased infiltration (Treatment 2), in comparison to the control animals.

Summary of the linear regression analysis:

Perform the analysis in R, using the following steps:

Read data into R, from an Excel spreadsheet or csv-file. From experience, the source data is often on the following (wide) format.

**Table:** 

Treatment	Tumor weight (g)	Marker 1 (Fl/mg)	Marker 2 (Fl/mg)	Marker 3 (Fl/mg)
1	–	–	–	–
2	–	–	–	–
3	–	–	–	–

Code:

library(readxl)

 library(tidyverse)

 #change cell range to fit data

 data <- read_excel(filename, range = "A01:E03")

Switch to long format by pivoting the marker columns to a common marker column:

Code:

data_long <- data %>%

 pivot_longer(cols = 3:ncol(data), names_to = 'Marker')

**Table:** 

Treatment	Tumor weight (g)	Marker	Value (Fl/mg)
1	–	Marker 1	–
1	–	Marker 2	–
1	–	Marker 3	–
2	–	Marker 1	–
2	–	Marker 2	–
2	–	Marker 3	–
3	–	Marker 1	–
3	–	Marker 2	–
3	–	Marker 3	–

Perform linear regression for each marker, investigating the effect of Treatment on Value, controlled by Weight. Compare the contrasts for each marker.

Code:

marker_list <- sort(unique(data_long$Marker))

 for(i in seq_along(marker_list))

 {

 m = lm(Value ∼ Weight∗Treatment, data = data_long)

 m.lst <- lstrends(m, "Treatment", var="Weight")

 sigs <- pairs(m.lst)

 x <- as.data.frame(sigs) %>%

 select(c(contrast, p.value)) %>%

 mutate(Marker = marker_list[i])

 print(x)

}

(Optional) Visualize the relationships per marker.

Code:

ggplot(data_long, aes(x=Weight, y=Value, color = Treatment)) +

 geom_line() +

 facet_grid(Marker∼Treatment, scales = "free_y")+

 labs(y = "Events per mg tumor", x = "Tumor weight (g)")

## Limitations

The gating strategies described in this protocol enable detection of a broad range of immune cell populations. Depending on the research question, more markers may be added to the panels to detect specific additional populations.

Murine polymorphonuclear leukocytes (PMNs) are distinguished by gating on cells that are CD45^+^ CD11b^+^ Ly6G^+^ Ly6C^low.^^10^ PMNs comprise several myeloid immune cell populations that are capable of both inhibiting and promoting tumor cell activity. Examples of PMNs include Neutrophils and Polymorphonuclear myeloid-derived suppressor cells (PMN-MDSCs).^10^ PMN-MDSCs are known to suppress lymphocytes and promote tumor growth. Classical neutrophils on the other hand, can be either inflammatory or tumor promoting, depending on the tumor microenvironment.^5^^,^^10^ In the myeloid panels presented in this protocol, we don't distinguish between different subsets of PMNs. If it is of interest to do so, an antibody that detects CD14 can be added to the myeloid panels. In mice, classical neutrophils are defined as CD14^-^ PMNs, PMN-MDSCs are CD14^intermediate^ PMNs and activated PMN-MDSCs are CD14^high^ PMNs.^11^

In the tumor infiltrating myeloid panel we use CD86 as a marker of M1-like vs. M2-like macrophages. M1-like macrophages are CD86^+^ CD206^-^ while M2-like macrophages are CD206^+^ CD86^-^. Hence, CD206 can be added to the panel if wanted.^12^^,^^13^

The tumor infiltrating lymphocyte panel described here allows for quantification of general lymphocyte populations, such as B cells, NK cells, CD4 T cells and CD8 T cells, and a minimal classification of differentiation status of T cells, including memory, effector or naïve phenotypes and regulatory T cells. It is possible to do a much more extensive classification of each of these lymphocyte populations, depending on the particular interest, including but not limited to B cell differentiation status,^14^ NK cell differentiation and function,^15^ CD8 T cell effector function and stem-like versus exhausted phenotypes.^16^

In secondary lymphoid tissues, T_FH_ cells are important for B cell maturation, class switching and immunological memory.^17^ In the lymphoid panel presented in this protocol, we did not include T_FH_ cells. However, if this population is of interest, it can be distinguished by adding an antibody against CXCR5 to the panel. T_FH_ cells are CD45^+^ CD3^+^ B220^-^ CD4^+^ CD8^-^ PD1^+^ CXCR5^+^.^18^

Secondary lymphoid tissues are the center of lymphocyte activation, cross-presentation and class switching and are crucial for immunological responses and development of immunological memory. Different types of secondary lymphoid tissues are structured differently depending on physical differences. A lymph node filters lymphatic fluid while the spleen filters blood and recycles iron and removes damaged and old blood cells. These functional differences require different macrophage populations in spleens and lymph nodes.^19^ These different populations have not been included in the panels of this protocol. Instead, we designed a panel that can be used in both lymph nodes and in spleens. If tissue specific macrophage populations are to be distinguished. We then recommend to expand the myeloid panel for secondary lymphoid tissue, and to divide it into a spleen myeloid panel and a lymph node myeloid panel.

For a lymph node myeloid panel, antibodies against F4/80 and CD169 can be added to the myeloid secondary lymphoid tissue panel to distinguish between Subcapsular sinus macrophages (CD45^+^ CD11b^+^ CD169^+^ F4/80^-^), Medullary sinus macrophages (CD45^+^ CD11b^+^ CD169^+^ F4/80^+^) and Medullary cord macrophages (CD45^+^ CD11b^+^ CD169^-^ F4/80^+^).^20^

For a spleen myeloid panel, additional antibodies against F4/80, CD169, CD68, SIGN-R1 and MARCO can be added to the myeloid secondary lymphoid tissue panel. This will additionally identify Red pulp macrophages (CD45^+^ CD11b^-/low^ F4/80^high^ CD68^+^), Marginal zone macrophages (CD45^+^ CD11b^+^ F4/80^low^ CD169^-/low^SIGN-R1^+^ MARCO^+^) and Metallophilic macrophages (CD45^+^ CD11b^+^ F4/80^low^ CD169^+/high^SIGN-R1^-^ MARCO^-^).^21^^,^^22^ Ensure availability of a flow cytometer that can detect up to 14 different fluorophores or prepare a separate macrophage panel for spleens.

## Troubleshooting

### Problem 1

Red Blood Cell residues in the samples.

### Potential solution

Perform a second lysis step by repeating steps 36–37 of “tissue processing and digestion”.

### Problem 2

There are still many cell clumps in the tumor samples after digestion and Red Blood Cell lysis.

### Potential solution

Perform an additional DNase I wash, after step 35 in “tissue processing and digestion”, to break up the clumps: resuspend cell pellet in PBS supplemented with 100 μg/mL DNase I and incubate for 5 min at 18°C–25°C. Pipet up and down a couple of times at the end of the incubation period to break down the clumps.

### Problem 3

No immune cells due to too young or old mice.

### Potential solution

Mice older than 8 weeks and younger than 12 weeks at the start of the experiment are recommended.

### Problem 4

Lack of statistical power due to too small sample size.

### Potential solution

To achieve robust statistical significance, use a minimum of 5 animals per experimental group.

### Problem 5

Low cell viability.

### Potential solution

(1) Some tumor models are inherently more necrotic and have reduced immune cell viability compared to others. Despite this, optimized timing and workflow always benefit viability. If the cells have to “wait” for the next steps keep them on ice and, if possible, in Awesome Wash Buffer.

(2) During the staining steps, centrifuge at 350–400 × *g* for 3 min, instead of 350–400 × *g* for 5 min. Additionally, consider incubating with live/dead viability dye at 2°C–8°C instead of at 18°C–25°C. This will slightly reduce the staining intensity, but might improve viability for sensitive cell populations.

### Problem 6

FMO controls have a lot of background, or seemingly two populations with different levels of background.

### Potential solution

Possibly different samples have different levels of background, or more/less cells with a particular level of background. To solve this, make sure to generate separate FMO controls for each tissue type, and/or each treatment group.

### Problem 7

There are too few cells recovered in the desired cell population on the flow cytometer.

### Potential solution

Include the CD45-positive enrichment step (for tumor samples only). Additionally, or alternatively, stain and analyze a larger fraction of the sample, by increasing the staining volume of the antibody mix. For example, if you want to stain and analyze 1 × 10^8^ cells to collect enough cells in the desired cell population, stain in double the volume of 100 μL instead of 50 μL.

## Resource availability

### Lead contact

Further information and requests for resources and reagents should be directed to and will be fulfilled by the lead contact, Diana Spiegelberg (diana.spiegelberg@uu.se).

### Technical contact

Questions about the technical specifics of performing the protocol should be directed to the technical contacts, Katrine Ingelshed (Katrine.Ingelshed@igp.uu.se ) and Marit Melssen (Marit.Melssen@igp.uu.se).

### Materials availability

This study did not generate new unique reagents.

### Data and code availability

All data and code are available in the published paper.

## Acknowledgments

We would like to thank Amber N. Woods, Ashley L. Wilson, Robin S. Lindsay, and Victor H. Engelhard from the University of Virginia for their input in developing the tissue processing protocol. We thank Christer Malmberg for the help with data analysis. D.S. was financed by the Swedish Cancer Society (21 0371 FE and 24 3787 Pj), Swedish Childhood Cancer Fund (FT2023-0023 and PR2023-0111), and Erik, Karin, och Gösta Selanders Stiftelse and Åke Wibergs Stiftelse. M.M.M. was supported by a postdoctoral grant from the Swedish Childhood Cancer Fund (TJ2022-0016).

We also acknowledge the Biomedicum flow cytometry core facility and Karolinska Institutet for support. Graphical abstract, Figures 1 and 2, and Figures S1–S4 were created using BioRender.com.

## Author contributions

K.I., M.M.M., and D.S. conducted the experiments; K.I., M.M.M., and D.S. analyzed the data and wrote the paper; and D.S. supervised the project and conceptualized the study.

## Declaration of interests

The authors declare no competing interests.
