# Supplementary material for: Protocol for in vivo immune cell analysis in subcutaneous murine tumor models using advanced flow cytometry
Source: STAR Protoc. 2025 Jan 15;6(1):103505. doi: 10.1016/j.xpro.2024.103505 (PMC11786769; doi:10.1016/j.xpro.2024.103505)
Supplement: Document S1. Figures S1–S4 [file mmc1.pdf]

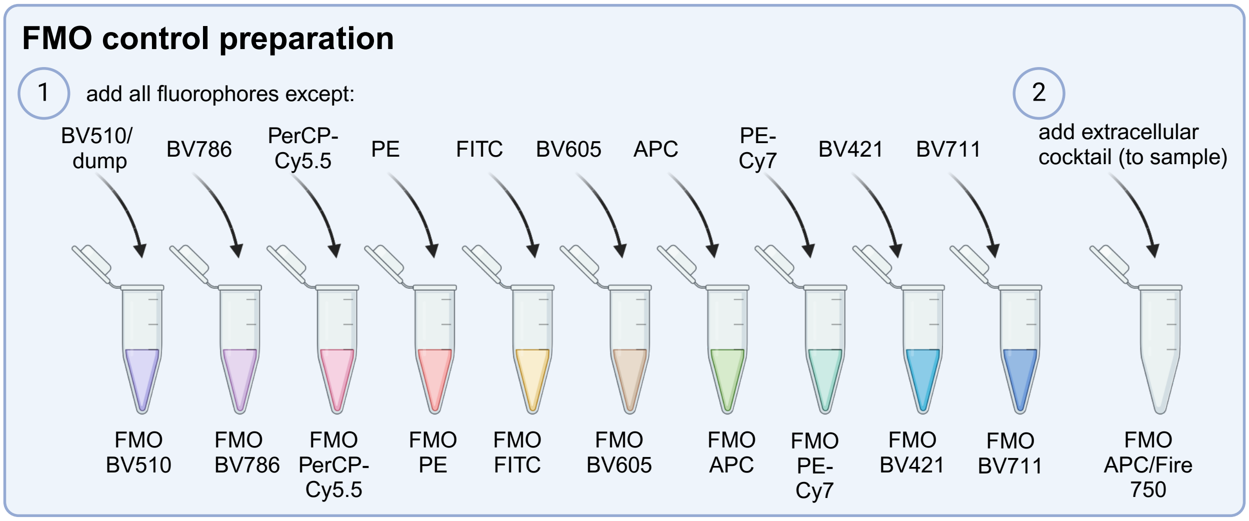

SI. Figure 1. Preparation of FMO controls (example), related to Step 8 and 15. To prepare the FMO controls, add all the antibodies in the panel except the one to control for, to the staining buffer. The lack of fluorescent signaling from the excluded antibody will serve as a negative gating control. Note that a separate FMO cocktail does not need to be prepared for the intracellular FMO control (APC/Fire-750 in this example). Instead, the extracellular antibody cocktail can be used to stain this FMO control, intracellular staining is instead excluded. The FMO panel example demonstrated here is based on the Tumor infiltrating lymphocyte panel (Table 3).

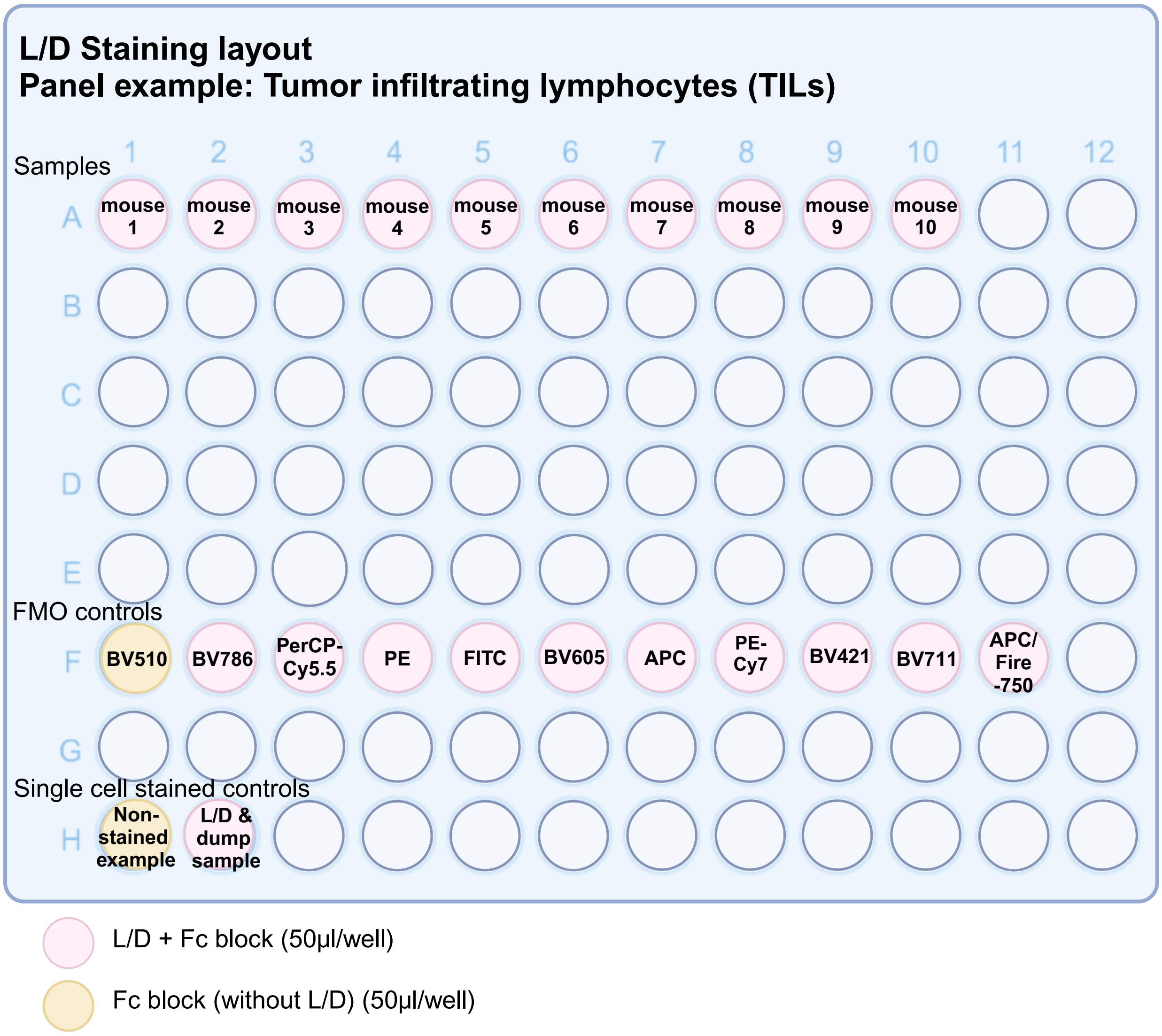

SI. Figure 2. Fc block and Live/dead staining example, related to Step 72 and 78. Add the L/D stain and Fc block to all samples and to all FMO controls except the BV510 FMO control (L/D and dump). Also add L/D and Fc block to the cell-based BV510 compensation control. Add only Fc block (without L/D stain) to the unstained cell-based compensation control (non-stained example) and to the BV510 FMO control. The FMO panel example demonstrated here is based on the Tumor infiltrating lymphocyte panel (Table 3).

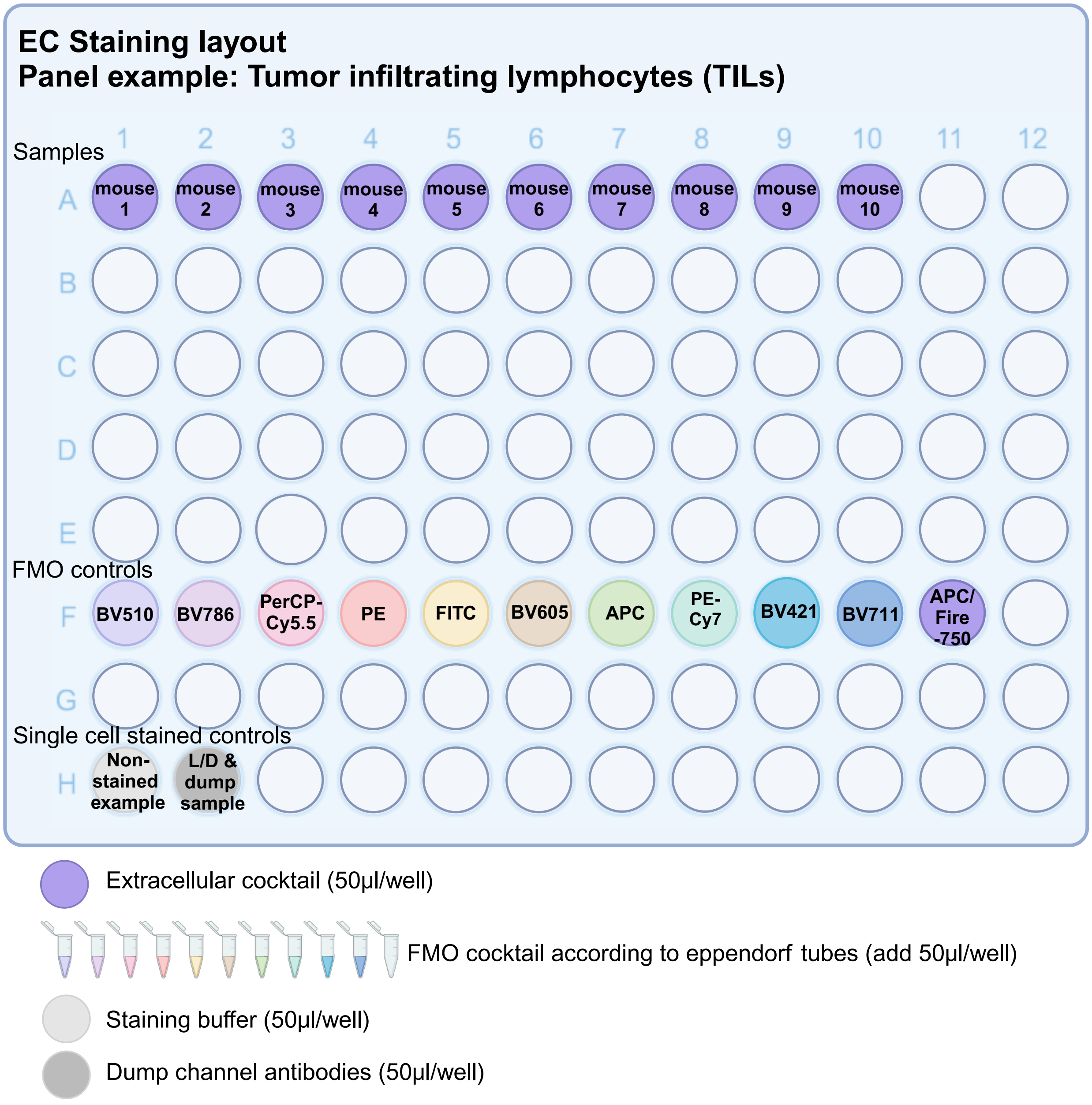

SI. Figure 3. Extracellular staining example, related to Step 80. Stain the samples and the intracellular FMO control (APC/Fire-750 in this example) with the extracellular cocktail. Add each prepared FMO cocktail (as prepared according to SI Figure 1) to each FMO control respectively. Add only stain buffer to the unstained cell-based compensation control (non-stained example). Add the cocktail of BV510 conjugated antibodies to the cell-based BV510 compensation control (L/D and dump control). The FMO panel example demonstrated here is based on the Tumor infiltrating lymphocyte panel (Table 3).

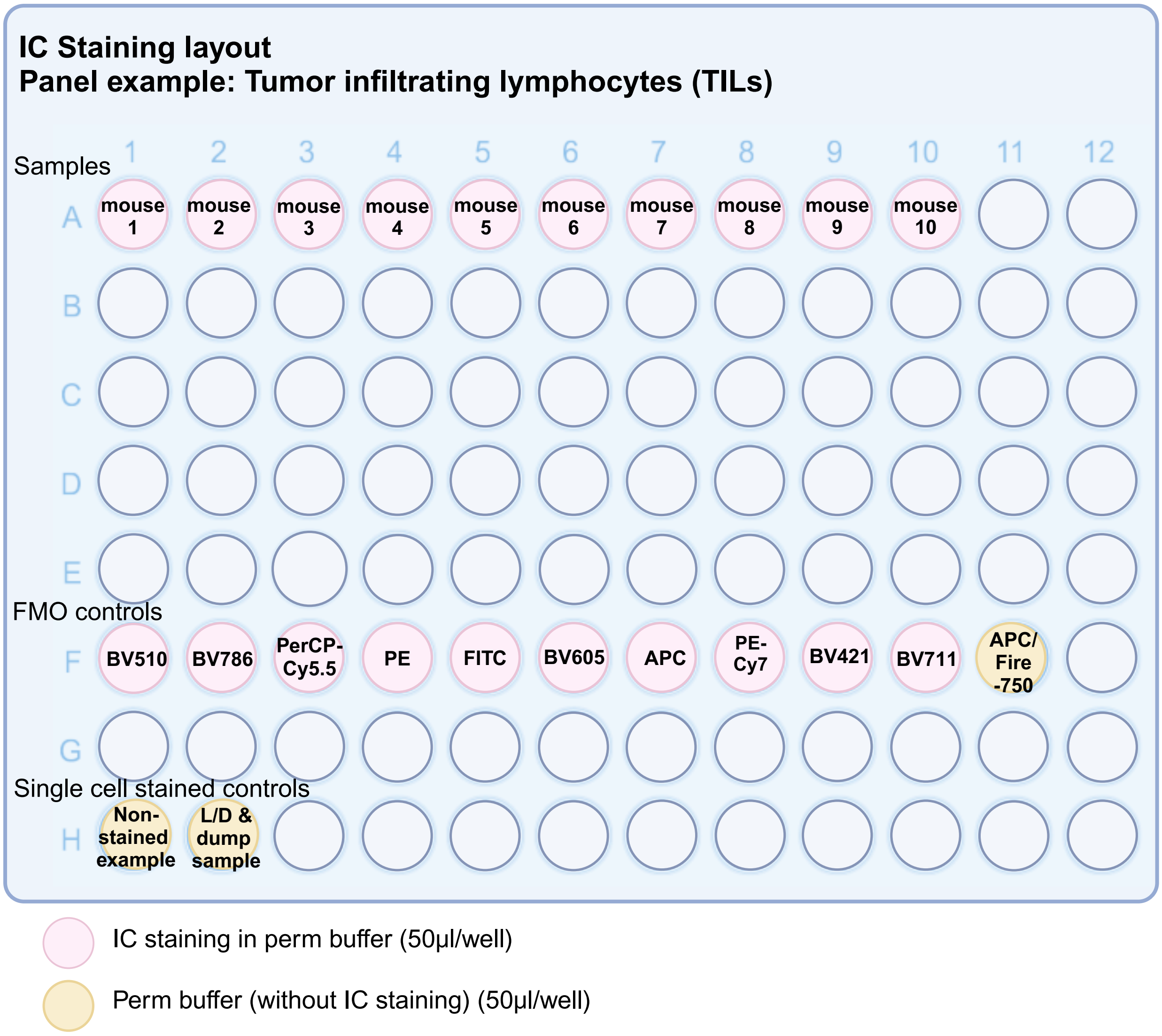

SI Figure 4. Intracellular staining example, related to Step 83. Add the intracellular antibody in perm buffer to all samples and to all FMO controls except the Intracellular FMO control (APC/Fire-750 in this example). Add only perm buffer to the Intracellular FMO control and to the cell-based compensation controls (non-stained and L/D and dump). The FMO panel example demonstrated here is based on the Tumor infiltrating lymphocyte panel (Table 3).
